# Preparation and Surface Functionalization of Carboxylated Cellulose Nanocrystals

**DOI:** 10.3390/nano11071641

**Published:** 2021-06-22

**Authors:** Edmond Lam, Usha D. Hemraz

**Affiliations:** Aquatic and Crop Resource Development Research Centre, National Research Council of Canada, Montreal, QC H4P 2R2, Canada; Edmond.Lam@nrc-cnrc.gc.ca

**Keywords:** carboxylated cellulose nanocrystals, functionalization, surface treatment, nanomaterial, biomaterial

## Abstract

In recent years, cellulose nanocrystals (CNCs) have emerged as a leading biomass-based nanomaterial owing to their unique functional properties and sustainable resourcing. Sulfated cellulose nanocrystals (sCNCs), produced by sulfuric acid-assisted hydrolysis of cellulose, is currently the predominant form of this class of nanomaterial; its utilization leads the way in terms of CNC commercialization activities and industrial applications. The functional properties, including high crystallinity, colloidal stability, and uniform nanoscale dimensions, can also be attained through carboxylated cellulose nanocrystals (cCNCs). Herein, we review recent progress in methods and feedstock materials for producing cCNCs, describe their functional properties, and discuss the initial successes in their applications. Comparisons are made to sCNCs to highlight some of the inherent advantages that cCNCs may possess in similar applications.

## 1. Introduction

Interest in biomass-based nanomaterials has grown in the last decade owing to their unique functional properties and sustainable resourcing. Currently, cellulose nanocrystals (CNCs) are among the leading biomass-based nanomaterials in terms of publications, applications developed, and technology readiness level (TRL). First produced by Nickerson and Habrle in 1947 [1], CNCs are crystalline, rod-shaped particles ranging in size from 5 to 20 nm in width and hundreds of nm in length, depending on the biomass source and production method. In order to obtain CNCs, native semi-crystalline cellulose is broken down into its elementary crystalline domains with the concurrent removal of amorphous cellulose segments. With its high aspect ratio [2], colloidal stability in aqueous media [3], liquid crystalline properties [4], and biocompatibility [5], CNCs have been used in a diverse range of applications, including but not limited to polymer composites [6], electronics [7], biomedical [8], and photonic films [9]. A number of excellent review papers have been published over the years that document the technological advances related to CNC’s research [10,11,12,13,14,15,16].

The most commonly used method for CNC’s synthesis is via sulfuric acid-mediated hydrolysis, in which removal of the amorphous cellulose segments is facilitated by the hydrolysis of glycosidic bonds and concomitant esterification of surface hydroxyl groups to form sulfate half-ester groups [17]. Mukherjee and Woods found that acid concentration (64 wt. % sulfuric acid) was an important factor in producing sulfated cellulose nanocrystals (sCNCs) [18]. Today, these sCNCs are produced by a number of companies, including Alberta-Pacific Forest Industries, GranBio, and the industry-leading Celluforce, who utilize forestry pulp feedstocks [15]. At the moment, the leading industrial producers of cCNCs are located in Canada: Anomera (30 kg/day production, target 1 ton/day in 2021; hydrogen peroxide-assisted) and Blue Goose Biorefineries (10 kg/day production; transition-metal-catalyzed) [15]. These daily production rates are only at the pilot scale and lag behind the industry leader, Celluforce, which has a production capacity of 1 ton per day for sCNCs. Although sulfuric acid hydrolysis is the predominant method for producing CNCs, there are many other methods reported in the literature that allow for the preparation and isolation of CNCs at various levels of TRL. These methods include enzymes [19], oxidizers [20,21,22], mechanical treatments [23], or a combination of these means [24,25]. Efforts in developing alternative CNCs’ production methods to sulfuric acid hydrolysis have been driven by the various challenges associated with using sCNCs in downstream applications, including low thermal stability [26] and nanomaterial aggregation [27].

Researchers have also turned to other methods to improve the economics and sustainability of the CNC production process. The sulfuric acid hydrolysis method is a harsh reaction due to the corrosiveness of the acid employed and favors the use of pure cellulosic feedstocks (forestry pulp materials, for example) to produce sCNCs. When biomass waste streams (such as raw lignocellulosic biomass containing cellulose, lignin, pectin, and other biopolymers) are used in the same process, it often leads to a low-yielding, impure CNC product that requires additional purification steps [20]. Alternative methods and different cellulosic feedstock materials have led to the production of CNCs with varying yields and different functional properties (morphology, surface chemistry, and surface charge density) compared to sCNCs. For example, other mineral acids such as HCl [28] and H_3_PO_4_ [29] have been used to produce CNCs, but these products have low surface charge and, as a result, do not disperse as well as the sCNCs. Carboxylated cellulose nanocrystals (cCNCs) have similar colloidal stability, uniform nanoscale lengths, and high crystallinity compared to sCNCs. Similar to sulfate half-ester groups of sCNCs, cCNCs possess carboxyl groups that promote the electrostatic repulsion between neighboring CNCs that prevents aggregation. One particularly enticing feature of cCNCs is the ability for these carboxyl groups to undergo further reactivity for surface modification to tailor the properties of the nanomaterial for downstream applications.

This review provides a summary of the different methods used to produce cCNCs from a diverse range of feedstock materials. With the availability of the carboxyl groups on the surface of the nanocrystal, a wide range of chemical modification approaches were explored to further alter the functional properties of the nanomaterial. As methods to produce cCNCs have become more mature, more applications utilizing cCNCs are emerging, and examples in the recent literature are also presented. Comparisons of cCNCs to sCNCs highlight the advantages the carboxyl group may bring to these end applications. Finally, a future outlook is provided to address future challenges and directions. This review is focused on the direct synthesis of cCNCs from biomass feedstocks and excludes reactions performed on nanocelluloses and other types of cellulose derivatives.

## 2. Production of cCNCs

**Scheme 1:**
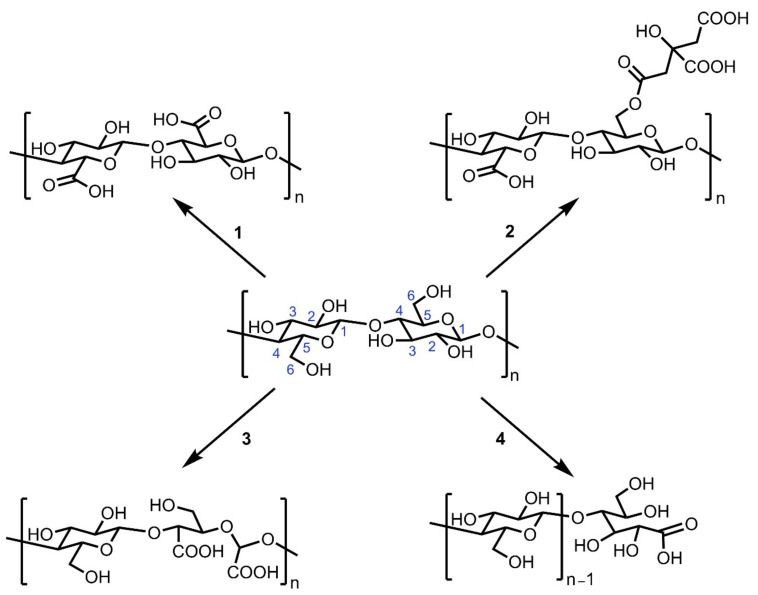
Four main types of cCNCs, produced through reactions on various cellulosic sources and utilized for a wide range of applications.

It is possible to obtain cCNCs through four general classes of reactions (Scheme 1), and although they all feature the carboxyl functional group on the surface of CNCs, the location of the carboxyl groups differ slightly:

1. Carboxyl group on the C6 position of the cellulose chain, generally obtained through oxidation of the primary hydroxyl groups at the C6 position.
2. Carboxylic acid group tethered to a moiety covalently attached to the hydroxyl group on the C6 position in addition to carboxylic acid group on the C6 position when considering two anhydroglucose units (AGU), typically obtained through the esterification of cellulose hydroxyl groups with carboxylic acids.
3. 2,3-Dicarboxylic acids from glucose ring opening.
4. Carboxylic acid groups found exclusively at the reducing ends of CNCs, which can be obtained due to the chemistry of the highly reactive aldehyde functional group.

### 2.1. Class 1: Carboxyl Group on the C6 Position of the Cellulose Chain

**Scheme 2:**
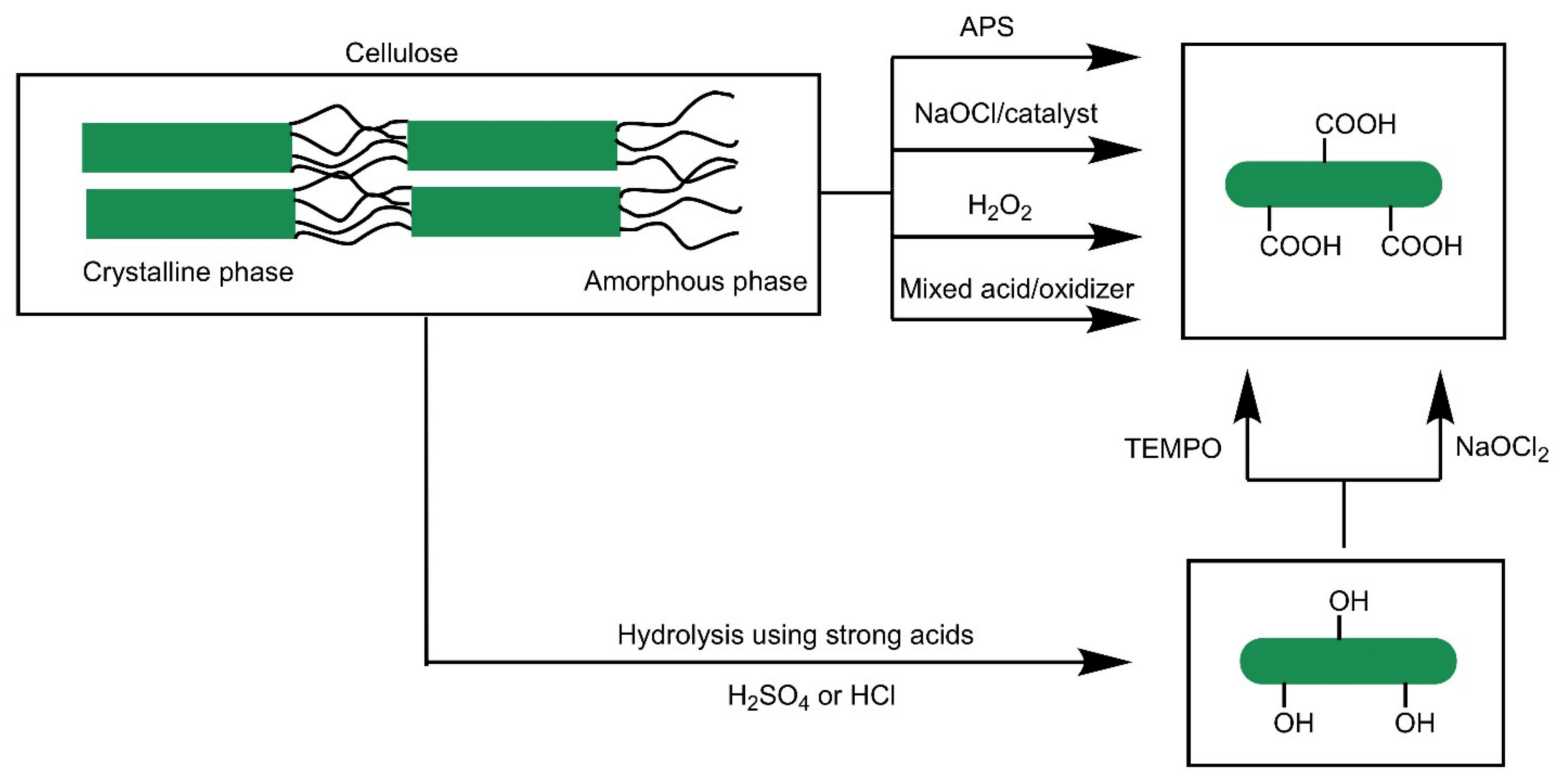
General methods for obtaining cCNCs from cellulosic sources.

Among the four classes of cCNCs depicted in Scheme 1, most of the widely used methods produce cCNCs containing carboxyl groups at the C-6 position. It is possible to generate this type of cCNCs either through various oxidative hydrolysis processes or through a two-step methodology of acidic hydrolysis, followed by oxidation of cellulosic biomass. The most commonly used oxidizing agents for the generation of cCNCs with carboxyl groups at the C-6 position are shown in Scheme 2.

#### 2.1.1. TEMPO

**Scheme 3:**
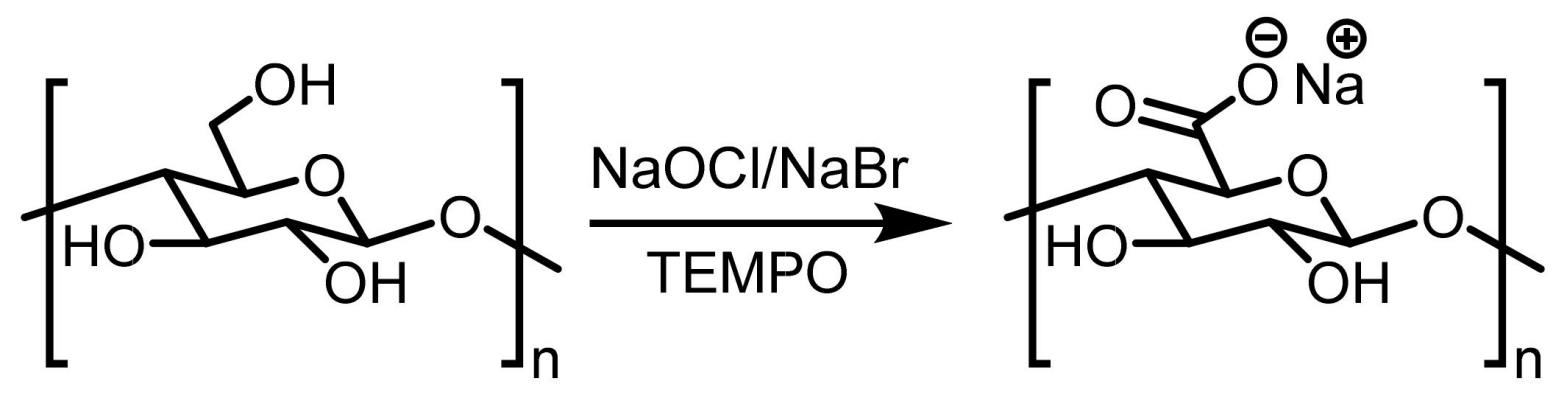
TEMPO oxidation of CNCs [22].

**Scheme 4:**
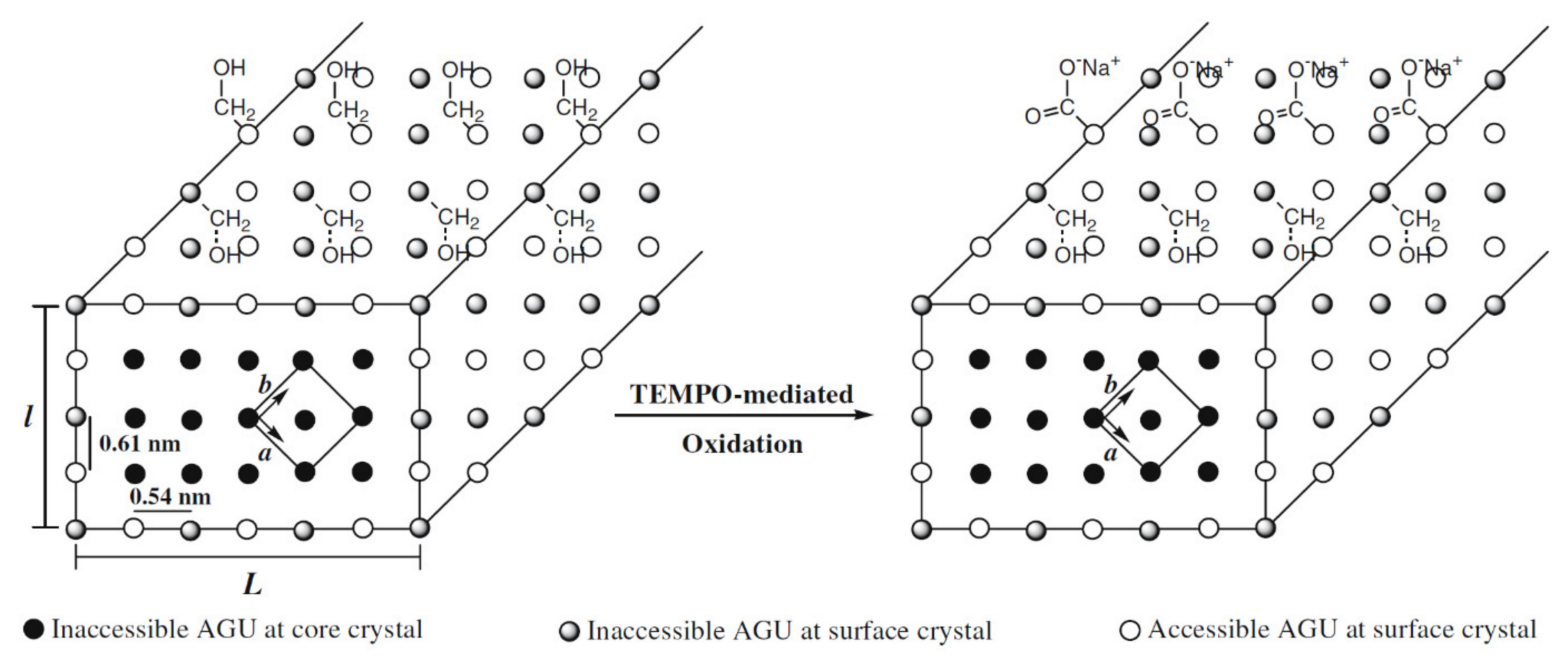
TEMPO oxidation of accessible hydroxyl groups at the surface of the CNCs (Reproduced with permission from [31]. Copyright Springer Science Business Media, Inc. 2006).

The 2,2,6,6-tetramethyl-1-piperidinyloxy (TEMPO)-mediated oxidation reaction is the most popular way to incorporate carboxylic acid groups on the surface of the nanocrystals (Scheme 3) through the oxidation of the primary hydroxyl groups of CNCs. In most cases, this method has been adopted as a post-production strategy on CNCs produced from the acid hydrolysis of cellulosic sources. The TEMPO oxidation was first introduced by De Nooy et al., whereby primary hydroxyl groups on polysaccharides were selectively oxidized in the presence of the secondary hydroxyl groups [30]. Its applicability on CNCs was first reported by Vignon and co-workers on nanocrystals produced from the HCl hydrolysis of cotton linter, subjected to a mixture of sodium hypochlorite, sodium bromide, and TEMPO for the selective oxidation of the primary surface hydroxyl groups to produce cCNCs. The carboxyl content or the degree of oxidation (DO) can be measured by conductometric titrations, FT-IR, and methylene blue adsorption, with conductometry being the most reliable method. In this case, a DO of 0.15 was obtained by conductimetry [22]. Due to the presence of negative surface charges, the cCNCs formed are well-dispersed in water and produce non-flocculating birefringent suspensions. It was noted, however, that excessive TEMPO oxidation led to a decrease in the crystal size. As such, the authors next optimized the oxidation process by varying the ratio of NaOCl to AGU such that only the accessible primary hydroxyl groups on the surface of the nanocrystals would be oxidized without affecting the core. The remaining hydroxyl groups are not affected by the oxidation process–this includes the inaccessible half of the primary hydroxyl groups on the surface, secondary hydroxyl groups on the surface, and all hydroxyl groups of the glucose units in the core crystal (see Scheme 4). This is consistent with other cCNCs of this class, where the carboxyl group is located exclusively on the C6 position of the cellulose chain. The TEMPO-oxidized product is often used as a precursor for further chemical modification via the reactive carboxylate group. While this review is not an exhaustive account of all TEMPO-mediated oxidation reactions and their subsequent transformations, some examples of further chemical functionalization and applications are discussed in later sections.

As opposed to using TEMPO oxidation to introduce carboxyl groups after CNCs’ production, Isogai and co-workers initially treated softwood bleached kraft pulp (SBKP) and microcrystalline cellulose (MCC) with a TEMPO mixture system [24]. After surface oxidation of the primary hydroxyl groups, the oxidized cellulose materials were exposed to cavitation-induced forces by sonication in water for 10–120 min to produce cCNCs with an average length of 200 nm and 3.5–3.6 nm in width. cCNCs prepared from SBKP exhibited higher mass recovery ratios and carboxylate content compared to those produced from MCC. Furthermore, the cCNCs prepared by TEMPO-sonomechanical treatments had a more uniform size distribution and exhibited a higher negative surface charge compared to CNCs prepared by acid hydrolysis, and did not require further purification through dialysis.

Faria and co-workers produced cCNCs from elephant grass by milling and hydrothermal processing with H_2_SO_4_ to remove hemicellulose and other extractives and NaOH to remove lignin [32]. This pre-treated feedstock was oxidized by TEMPO and mechanically disintegrated by sonication to yield cCNCs. They demonstrated that cCNCs are potential anti-biofouling agents that utilize contact-mediated membrane stress as the mechanism governing the toxicity of CNCs towards bacteria cells. In follow-up work, the same elephant grass cCNCs were crosslinked with polyamide membranes to produce antimicrobial thin-film composites [33].

#### 2.1.2. Ammonium Persulfate (APS)

The TEMPO process is a powerful reaction to convert surface hydroxyl groups of CNCs to carboxyl groups, and yet it has several limitations. To produce cCNCs, either acid hydrolyzed CNCs are subjected to a post-production surface modification with TEMPO [22], or TEMPO-oxidized cellulosic materials are further treated with acid hydrolysis [31,34] or sonomechanical treatments [24]. The TEMPO process cannot directly produce cCNCs from longer-chain cellulosic materials as it is unable to break down the amorphous domains of cellulose [35]. To this end, other non-acid methods have been sought for the production of CNCs with similar potency to mineral acids. Despite the low cost and high abundance of mineral acids like sulfuric acid, these production methods require expensive corrosion-resistant equipment, multiple treatments, and isolation steps such as dialysis. In 2011, Luong and co-workers developed a process to produce cCNCs using APS, a low-cost, low-toxicity oxidant, from a variety of native plant materials and bacterial cellulose [20]. The cellulosic materials were heated at 60 °C in 1 M APS for 16 h with vigorous stirring. Under these conditions, the persulfate anions undergo two reactions in solution:

S_2_O_8_^2−^ + heat → SO_4_·^−^ (1)

S_2_O_8_^2−^ + 2H_2_O → 2HSO_4_^−^ + H_2_O_2_ (2)

The free radicals and hydrogen peroxide are capable of hydrolyzing the amorphous cellulose, decolorizing the product via the oxidative breakdown of the aromatic components of the plant material, and oxidizing the C6 alcohol groups on the surface of the nanocrystal. The yields of the cCNCs vary greatly as they depend on the starting biomass materials: native plant materials (such as flax, hemp, and triticale) yield lower amounts of cCNCs compared to MCC or paper filters as the plant materials possess additional components such as lignin, hemicellulose, and wax, which lower the weight percentage of cellulose in the starting material. The DO of the resulting cCNCs ranged from 0.11 to 0.19. From TGA analysis, cCNCs produced were found to be more thermally stable than sCNCs. To better understand the nature of the APS reagent breakdown, Lam et al. conducted Raman spectroscopy studies on the reaction mixtures of APS and MCC and found that nearly 60% of the sulfate ions in solution were attributed to the H_2_SO_4_ [36]. By the addition of NH_4_OH to the solution, it was possible to not only initiate the recovery of sulfate anions as ammonium sulfate but the resulting neutralized cCNCs with COO^−^NH_4_^+^ groups exhibited better dispersion and thermal characteristics over cCNCs with COOH and COO^−^Na^+^ groups.

A limitation to the APS method by Luong and co-workers is the large amount of APS required for biomass conversion (22.8 g APS per g of biomass) and the long reaction time (minimum of 16 h) [20]. Liu and co-workers investigated the use of *N*,*N*,*N*’,*N*’-tetramethylethylenediamine (TMEDA) and ultrasonic-assisted disintegration to address the economic issues of the APS oxidation [37]. Under the conventional APS method, a single type of free radical is produced (SO_4_·^−^), which Liu et al. believe results in the relatively weak hydrolysis ability of APS, necessitating a higher loading of the oxidant and longer reaction times to produce cCNCs. By using TMEDA as a redox initiator, two additional free radicals (SO_4_·^−^ and HO·) were also produced that can react with the biomass at higher redox potentials [38]. An ultrasonic step was further added to the process to promote disintegration of the cellulosic chain by increasing the surface area for increased reaction sites. Under these conditions, cotton pulp was successfully converted to cCNCs in 6 h at 75 °C for a yield of 62.5%, with lower consumption of APS (8.5 g APS per g of pulp).

Amoroso et al. examined the use of a microwave-assisted process to overcome the inefficiencies of heating currently used in the conventional APS process [39]. They found that controlling the heating ramp time and holding the heating time were important parameters to allow for sufficient time for the APS to convert to the active radical oxidant reacting with the biomass. Limiting microwave power output also reduced the potential of carbonizing the cellulosic material, which was detrimental to the cCNC’s yield. Cotton residues were converted to cCNCs in 90 min under microwave-assisted heating, which demonstrates the process as an effective way to reduce the reaction time of the APS process. However, the limitation to the microwave-assisted reaction comes from scalability: APS conventional heating can be performed at 1000 L, while the current microwave-assisted method has only been demonstrated at 50 mL volume.

Researchers are often interested in comparing hydrolysis methods for producing CNCs from a variety of waste streams, whether they are post-consumer products, agricultural residues, or unlikely non-plant sources of cellulosic materials. For example, recycled paper waste is often viewed as a source of secondary cellulosic fibers for valorization. Jiang et al. evaluated the conversion of these materials into sCNCs and cCNCs (by APS oxidation) [40]. cCNCs were produced in lower yield (22.42%) compared to sCNCs (41.22%), with a crystallinity index (CrI) of 77.56% and 72.45%, respectively. It was noted that sulfuric acid residues on the surface of the sCNCs could promote the carbonization of cellulose and increase the production of carbon residues. The carbon residue of sCNCs was 26.73% compared to only 14.17% for cCNCs. Zhang and co-workers investigated different methods to produce CNCs from lemon (*Citrus limon*) seeds, including sulfuric acid treatment for sCNCs, and both APS and TEMPO oxidation for cCNCs [41]. In all three methods, the CNCs retained their cellulose Iβ structure. TEMPO-oxidized cCNCs produced the highest yield of cCNCs with larger nanorod dimensions (~360 × 34 nm) and lower CrI compared to the other two methods. APS-treated cCNCs had the highest CrI and sCNCs the smallest nanocrystal dimensions. The lemon-seed-derived CNCs were tested in Pickering emulsions, in which the sCNCs and APS-treated cCNCs exhibited the best stabilizing effects compared to the TEMPO-oxidized cCNCs.

The use of APS to convert bamboo borer powder (produced when *lyctus bruneus*, a beetle, attacks the woody bamboo materials to yield a flour-like powder) to cCNCs as a means to valorize bamboo raw materials was investigated [42]. The resulting cCNCs were more spherical in shape (20–50 nm) and had a CrI of 62.75%. The spherical geometry of these particles could be attributed to the nanoparticles and was possibly created by a self-assembly process of CNCs and their fragments from the less-crystalline cellulose sources [43]. Lu and Hsieh also produced spherical sCNCs from chardonnay grape skins in which the starting cellulose’s CrI increased from 54.9% to 64.3%. The authors believe that the lower crystallinity of the grape skin cellulose produces fewer rod-like crystals compared to cotton and wood, which have larger intact crystalline regions. Furthermore, the drying process could promote the aggregation of smaller, more abundant, non-rod nano-fragments around less abundant, larger nano-rods by hydrogen bonding, leading to a core–shell spherical cellulose nanostructure.

Tunicates are marine invertebrate sea animals that are the only known animal source of cellulose. Cellulose from the mantle of *Halocynthia roretzi* was extracted through several alkali treatments and bleaching processes. The tunicate cellulose was then treated with APS and ultrasonic post-processing to produce cCNCs [44]. The formation of tunicate cCNC lyotropic chiral nematic liquid crystals was observed for the first time, which displayed birefringence and a fingerprint texture. The critical concentration of phase separation for tunicate cCNC suspension was around 3.5 wt%. Solid films were subsequently prepared from evaporation-induced self-assembly, which showed preservation of the chiral nematic structure of tunicate cCNCs. In previous work using TEMPO-oxidized tunicate CNCs, only non-uniform birefringence was observed, which may be attributed to the high polydispersity of the length (from 0.1 to 10 μm) of the tunicate CNCs and their high viscosity suspensions [31].

Pan and co-workers converted bleached kraft pulp (cellulose I feedstock) into cellulose II using a mildly acidic lithium bromide trihydrate (MALBTH) system to induce the partial hydrolysis and polymorph transition [45]. APS was then used to convert the cellulose II into cCNCs II, which were found to be smaller in size compared to those of cellulose I cCNCs that were not subject to the polymorph transition procedure. Other researchers have focused on different feedstocks to produced cCNCs by the APS method, including recycled medium-density fiberboard [46], denim waste [47], and balsa and kapok fibers [48].

#### 2.1.3. Hydrogen Peroxide

One of the simplest and greenest oxidizing agents available is hydrogen peroxide. Anomera, a company based in Quebec, Canada, has developed a process to produce cCNCs in one step from biomass, dissolving pulp and wood waste using 30% hydrogen peroxide at 115 °C for about 8 h using a method developed by Andrews and Morse [21]. An advantage to this process is the full consumption of hydrogen peroxide. In the same patent, it was also possible to produce cCNCs from spruce fibers at room temperature by irradiating a 30% hydrogen peroxide solution with UV light for 12 h. In addition, they were able to convert the negatively charged cCNCs to a positively charged nanomaterial by mixing the cCNCs with polydiallyldimethylammonium chloride (PDDA), a cationic polymer that is used as a wet-end additive in papermaking. The company’s current cCNC product, Dextracel, has a size of 150–250 nm in length, a width of 5–10 nm, crystallinity of >85%, and a carboxyl content of 0.12–0.20 mmol/g.

#### 2.1.4. Sodium Hypochlorite (NaOCl)/Sodium Chlorite (NaOCl_2_)

Blue Goose Biorefineries is a company in Saskatchewan, Canada, that produces cCNCs from a variety of biomasses using a transition metal-catalyzed oxidation process [49]. The process is characterized by three steps. In the first step, a redox reaction using sodium hypochlorite and a catalyst (either iron or cupric sulfate) is employed to break down the starting biomass. The isolated biomass filter cake is then subjected to a sodium hydroxide treatment as part of the second step. In the final step, the alkaline-treated biomass is exposed to a second redox reaction in conditions similar to the first step, which finally results in the production of cCNCs. The yields of the reaction are dependent on the biomass source used. For example, the highest yields were reported for A96 high purity cellulose at 38.8% yield, but only 9.7% yield was reported for Yreka, a medium-density fiberboard with high lignin content. Despite similar reaction times required for all biomass materials tested, the amount of sodium hypochlorite required to produce cCNCs using this method increases with the lignin content in the starting biomass. The company’s current cCNC product, BGB Ultra, has a size of 100–150 nm in length, a width of 9–14 nm, crystallinity of 80%, and a carboxyl content of 0.15 mmol/g.

The pilot-scale nanocellulose production at USDA Forest Product Laboratory utilizes a combination of sulfuric acid, sodium chlorite, and 4% hypochlorite solution to produce 25 kg of cCNCs per batch, with nanocrystals of 5–20 nm in width and 150–200 nm in length [50]. Using a 50 kg machine-dried pre-hydrolysis kraft rayon-grade dissolving pulp into a 400 L reactor, the cellulose strips are subjected to sulfuric acid hydrolysis at 45° C for 90 min (300 L, 64 wt. %) under a nitrogen atmosphere. Upon quenching the reaction, the acidic suspension is treated with sodium chlorite, sodium hydroxide, and 4% hypochlorite solution for neutralization and bleaching of the nanocrystals, which had been discolored by the sugar degradation during the acid hydrolysis process. Carboxyl groups are likely introduced when the suspension is treated with sodium chlorite and hypochlorite solutions.

#### 2.1.5. Mixed Acid Solutions with Oxidizers

Some researchers have turned to using mixtures of acids and oxidizers to produce cCNCs. This strategy is intended to overcome some of the drawbacks that each reagent may contribute to the synthesis of the CNC product. For example, strong acids like sulfuric acid are capable of hydrolyzing cellulose to form CNCs, but the introduction of the sulfate ester groups may be undesirable as it may limit the utility of the CNCs by decreasing their thermal stability. Weak acids and oxidizers can be used to tune the surface chemistry of the nanocrystal but are unable to generate sufficiently high proton concentration to induce cellulose hydrolysis.

A one-pot procedure for cCNCs from cotton pulp was developed using 1% sulfuric acid, with potassium permanganate and oxalic acid, as the oxidizing and reducing agents, respectively [51]. cCNCs prepared from this method were 150–300 nm in length and 10–22 nm in width with a carboxyl content of 1.58 mmol/g. The authors believe that the oxalic acid can complex Mn^3+^ to form [Mn(C_2_O_4_^2−^)]^+^ and prevent the Mn^3+^ from being reduced to Mn^2+^, leading to the prolonged strong oxidizing capacity of the reaction system. Compared to sCNCs, their cCNCs’ solutions of >6 wt. % displayed chiral nematic liquid crystalline phases.

A one-step hydrolysis process to convert MCC to cCNCs was developed using sulfuric acid and nitric acid in 0.5 h, whereby the resulting cCNCs had physicochemical characteristics consistent with those obtained from APS and TEMPO oxidation [52]. From 50 to 90 °C, the length and width of the nanocrystals decreased in size and yield while exhibiting increasing DO (up to a maximum of 0.11 at 80 °C). High crystallinity for these cCNCs (average CrI about 90%) was reported, though the starting MCC CrI was already at 85.3%.

### 2.2. Class 2: Carboxylic Acid Group Tethered to a Moiety at C6 Position

While sulfuric acid is the most common mineral acid employed for the production of CNCs, its use comes with economic and sustainability challenges. For example, in producing 1 kg of sCNCs, nearly 9 kg of sulfuric acid is consumed, leading to the generation of 13 kg of Na_2_SO_4_ from acid neutralization, with only moderate yields reported (30–50%) [53]. Organic acids, specifically carboxylic acids, are gaining traction as alternative hydrolysis reagents to mineral acids to overcome these economic, technical, and environmental challenges. However, a key criterion to produce CNCs is to use organic acids with sufficient acid strength (pKa = 1–3). For example, formic acid (pKa = 3.77) was used to hydrolyze birch pulp, resulting in mainly micron-length cellulosic fibers [54]. Only after increasing the acidity of the reaction mixture with 2% HCl were CNCs of <1000 nm produced. In addition, the use of formic acid did not produce cCNCs; a subsequent TEMPO oxidation step was required to convert micron-length cellulosic fibers into cCNCs.

Chen et al. demonstrated the use of different dicarboxylic acids (oxalic and maleic acid) on a bleached eucalyptus kraft pulp to produce CNCs and cellulose nanofibrils [53]. The carboxylation of the cellulose is not a direct oxidation of the C6 alcohol of cellulose (as in the case of TEMPO or APS oxidation), but a Fischer esterification, in which the organic acid is added onto the cellulose. The use of organic acids of relatively lower acid strength (pKa = 1.25 for oxalic acid; pKa = 1.9 for maleic acid) led to the production cCNCs of longer length and greater thermal stability (albeit at lower yields) compared to those produced from sulfuric acid, which has a higher acid strength (pKa = −3.0). In the optimal case of using 70% oxalic acid at 100 °C for 60 min, only a 25% yield for cCNCs was reported. The majority of the cellulosic residue left in the reaction mixture could be processed by mechanical fibrillation to produce CNF. Due to the low solubility of the organic acids in water, the acid was easily recovered by crystallization; nearly 95% of the oxalic acid used in the reaction was recovered from the hydrolysate, reflecting a greener process.

**Scheme 5:**
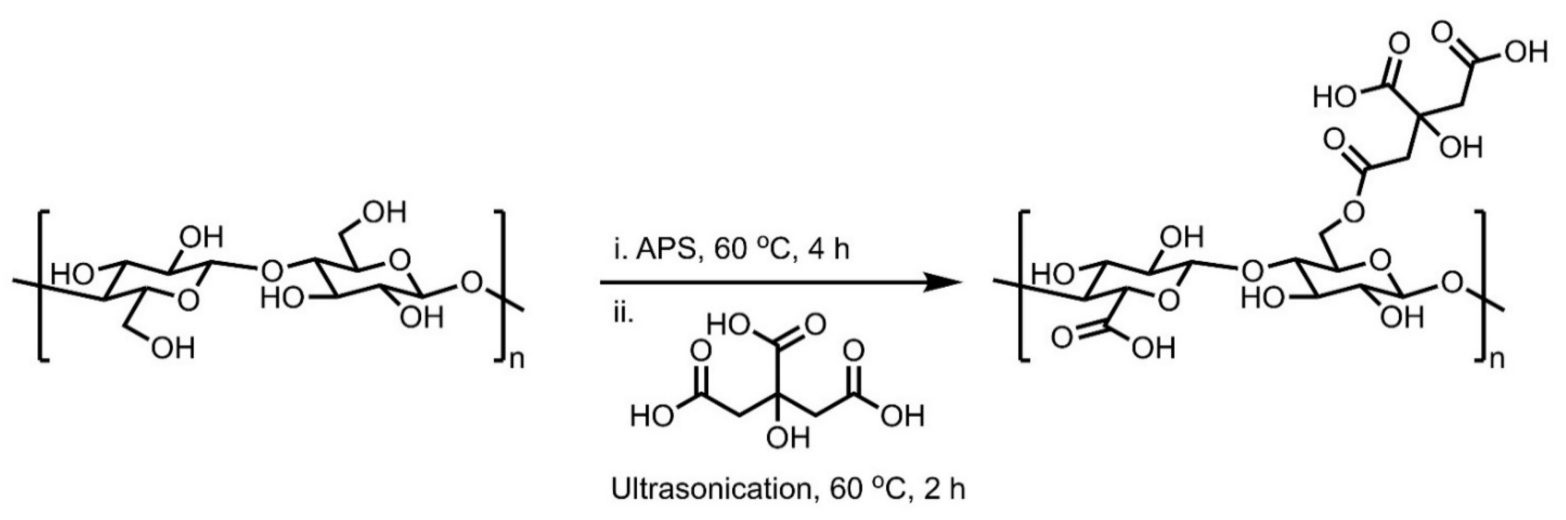
Reaction of microcrystalline cellulose with APS and citric acid to produce CNCs with dicarboxylic acid moieties [57].

An extension into weak tricarboxylic acids was reported by using citric acid and ultrasonication methods to produce cCNCs from sugarcane bagasse pulp [55]. The addition of the post-ultrasonication method increased the yield of the cCNCs by 21.6% in comparison to the non-ultrasonication protocol. cCNCs were recovered by dialysis and centrifugation steps, and the remaining oxidized cellulosic residues were subjected to homogenization to produce cCNF (63.4%). Citric acid was subsequently recovered from the reaction liquor by rotary evaporation. A common problem cited with the use of citric acid is the low yield of cCNCs obtained due to the weak acidity of citric acid (pKa = 3.13), which is corroborated by the much higher isolated yield of cCNF compared to cCNCs. Higher yields (87.8%) of cCNCs were reported using 90% citric acid and 10% HCl to convert MCC to cCNCs, but this process required the addition of the strong mineral acid HCl to promote hydrolysis [56]. These citric-acid-oxidized cCNCs were then used in the preparation of oil/water emulsions for food applications. Emulsions made with higher concentrations of cCNCs (5%) resulted in smaller droplet sizes (1.01 μm) compared to those produced at lower concentrations of cCNCs (0.1%) at an average droplet size of 17.8 μm. The smaller droplets were more effective in stabilizing the soybean oil over 28 days. A similar approach (Scheme 5) has been utilized to produce dicarboxylated CNCs using the oxidative hydrolysis of MCC by APS, followed by esterification with citric acid in the presence of ultrasonication [57].

An underutilized plant in African nations is the cellulose-rich Juncus plant (*Juncus effuses*). The Juncus plant has a grass-like structure consisting of hollow cylindrical rods about 1 m in height, and 4–8 mm in diameter and are typically harvested as materials for woven textiles. Kassab et al. investigated the production of CNCs from the Juncus plant [58]. Purified cellulose microfibers (CMF) were first obtained from the plant stems, and subsequent treatment with citric acid/HCl hydrolysis produced cCNCs with a length of 352 ± 79 nm, a diameter of 6.1 ± 2.8 nm, and a CrI of 83% with cellulose I structure. The authors speculated that the Juncus plant cCNCs could be used as a nano-reinforcing agent for polymer composites due to the high degradation temperature (231 °C). In another report, the authors evaluated the use of post-harvest tomato plant residue (TPR) as a sustainable source for the extraction of cellulose derivatives, namely, CMF and CNC [59]. After obtaining CMF with an average diameter of 20 μm from the TPR, the CMF was then treated with a mixture of citric acid and HCl to produce the cCNCs. The resulting TPR cCNCs also possessed a cellulose I structure with a length of 514 ± 131 nm, a diameter of 4.7 ± 1.4 nm, and a CrI of 78%. A higher degradation temperature of 243 °C was reported for the TPR cCNCs compared to sCNCs and phosphorylated CNCs produced in the study (219 and 235 °C, respectively), a trend that was also observed for the Juncus plant CNCs.

Liu et al. were able to increase the cCNC’s yield to 80.3% by using catalytic amounts of FeCl_3_ to enhance citric acid hydrolysis efficiency [60]. The authors believe that FeCl_3_ can promote the reaction in several ways: (1) as a Lewis acid to increase the acidity of the citric acid solution by polarizing the water molecules around the central metal ions, (2) the Fe^3+^ can coordinate with glucose to produce an intermediate complex with weakened C-O-C bonds that are more susceptible to bond breakage, and (3) disrupt inter- and intramolecular hydrogen bonds within cellulose to enhance the hydrolysis reaction.

### 2.3. Class 3: 2,3 Dicarboxylic Acids from Glucose Ring Opening

**Scheme 6:**
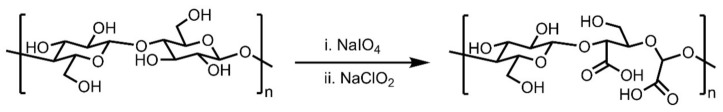
Formation of cCNCs via oxidative cleavage of C2–C3 bonds using sodium periodate oxidation, followed by oxidation of the aldehyde groups into carboxyl groups using sodium chlorite [61].

Carboxylation can be achieved by using sodium periodate, which can cleave the C2–C3 bonds of β-D-glucose monomer units of cellulose, and selectively oxidize C2 and C3 vicinal hydroxyl groups to form 2,3-dialdehyde units along the cellulose. These aldehyde groups can undergo further oxidation to form 2,3-dicarboxyl groups by sodium chlorite in an aqueous acidic medium (Scheme 6). This two step-process was used to convert softwood pulp into cCNCs [61]. Initial separation of the two fractions yielded mainly oxidized microfibrils. Upon alcohol addition, a second fraction of cCNCs was obtained with dicarboxylated functionalities on the surface of the nanomaterial and a carboxyl content ranging from 3.60 mmol/g to 6.60 mmol/g. Based on dynamical light scattering (DLS) measurements, these dicarboxylated cCNCs had significantly larger hydrodynamic radii, which the authors attribute to the presence of dicarboxylate chains protruding off the main CNC nanorods, contributing to the increased carboxyl content values obtained. Upon further hydrolysis of these dicarboxylated cCNCs, the carboxyl content of the materials decreased to 1.4 mmol/g as these dicarboxylate chains are more readily hydrolyzable compared to the crystalline CNC nanorods.

Sugarcane bagasse was used to prepare cCNCs using two methods: a single-step sodium periodate oxidation method and the two-step sulfuric acid/TEMPO oxidation method [62]. One aspect of the investigation was to compare the physicochemical properties of the cCNCs of the different oxidation methods. When the sodium periodate reaction was performed at room temperature, rod-like cCNCs (104 × 6 nm) were obtained. However, at 60 °C, spherical nanoparticles (approx. 24 nm in diameter) were formed instead, which the authors attribute to agglomeration of the crystalline regions of the cellulose. Acid hydrolyzed cCNCs showed greater crystallinity (69.0%) compared to either the rod-like (46.7%) or spherical (43.6%) cCNCs obtained from the sodium periodate method, which cleaves the chemical bond between the C2 and C3 positions, and retains regions of oxidized amorphous cellulose.

### 2.4. Class 4: Presence of Carboxylic Acid Groups at the Reducing End of CNCs

**Scheme 7:**
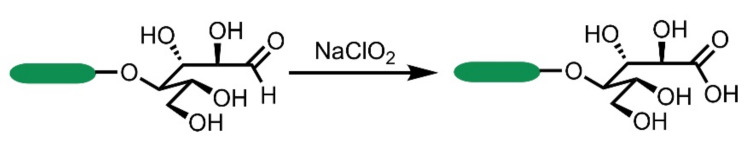
Oxidation of aldehyde groups at the reducing ends of CNCs using sodium chlorite [63].

The presence of the highly reactive aldehyde functional group at the reducing end of CNCs allows for the production of cCNCs through another method. CNCs are made up of β-1,4 linked anhydro-D-glucose units. Similar to the structure of cellulose, they have three components: the non-reducing end, the cellobiose sequence (the repeat unit made up of two glucose units), and the reducing end. The latter is a hemiacetal, which in its open-chain form contains an aldehyde functional group. As such, these aldehyde moieties can be selectively oxidized to produce carboxyl groups at the reducing ends of the nanocrystals. Hieta et al. successfully applied a method involving the use of sodium chlorite at a pH of 3.5 (adjusted using acetic acid) for 20 h at room temperature to produce microfibrils and nanocrystals with carboxyl groups at the reducing ends of the materials. The selective deposition of silver at the reducing ends served as a tool to elucidate mechanistic and structural aspects related to the parallel configuration of cellulose I [63]. This method was later reproduced to yield thiolated CNCs using thioethanolamine via an amide linkage [64]. The aldehyde groups at the reducing ends were then oxidized to produce the carboxylic acid derivative using sodium chlorite (Scheme 7). The CNCs used were from sulfuric acid hydrolysis and were therefore devoid of surface carboxyl groups prior to treatment with sodium chlorite. This type of cCNCs is very useful for selective functionalization at the end of the nanocrystals.

### 2.5. cCNCs from Different Feedstocks

Although commercial production of CNCs primarily uses cellulose-rich sources such as wood pulp, many researchers have turned to other feedstock materials that are either more regionally plentiful, or there is an imminent need to valorize specific biomass waste streams that would only be discarded into the environment if unused (such as agricultural residues). A key challenge to using any new biomass material is the weight percent content of cellulose versus its other components (i.e., lignin, hemicellulose, wax, and pectin) can vary from feedstock to feedstock. Table 1 summarizes some of the cCNCs previously discussed in Section 2, produced from different feedstock materials with a diverse range of physicochemical properties dependent on the method used. For the majority of the entries in Table 1, the feedstocks used to produce the cCNCs had undergone bleach processing prior to hydrolysis. Only the reactions utilizing APS were capable of hydrolyzing raw biomass into cCNCs.

## 3. Chemical Modifications of cCNCs

The presence of carboxyl groups on the surface of the nanocrystals not only provides electrostatic stabilization but also opens up a wide range of surface modification possibilities. It is possible to modify the surfaces of cCNCs through covalent and noncovalent functionalization. Compounds containing amino and hydroxyl groups can react covalently with the carboxyl moieties of the nanocrystals to form a wide range of derivatives. Similarly, it is possible to decorate the periphery of the nanocrystals with positively charged ions, polymers, or surfactants using noncovalent surface modifications, which rely on the electrostatic interactions with the negatively charged carboxylate groups. These modifications broaden the scope of applications of these nanomaterials by altering the surface properties and improving their dispersibility in non-aqueous media since unmodified cCNCs have limited dispersion in a non-aqueous environment, leading to flocculation.

### 3.1. Amidation

**Scheme 8:**
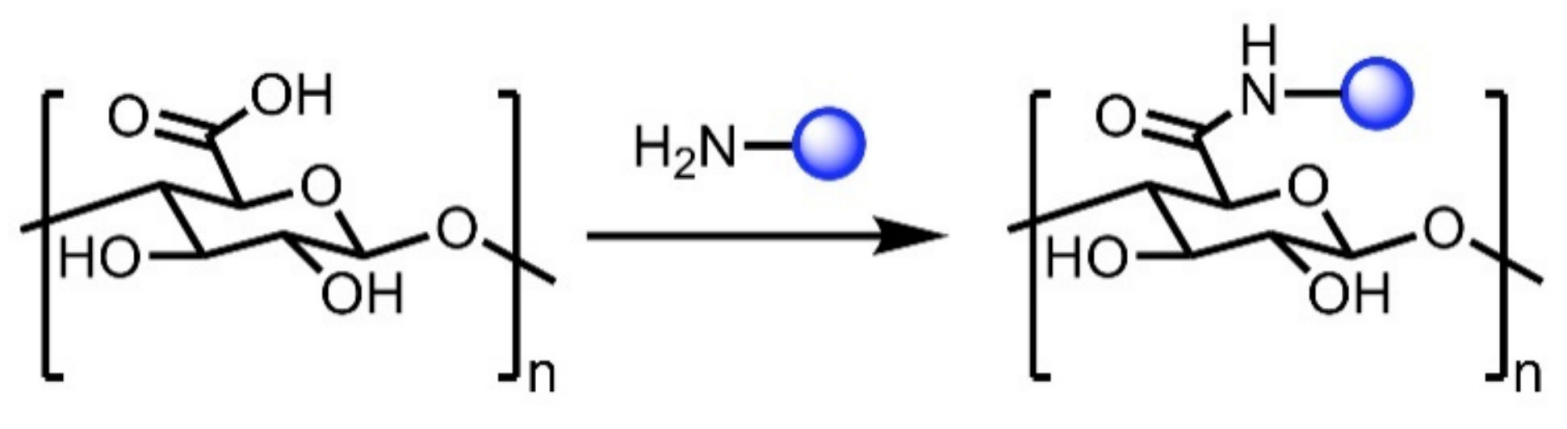
General amidation reaction of cCNCs.

The most commonly used covalent reaction that cCNCs undergoes is the amidation reaction, which relies on the strong reactivity of the carboxyl and amino groups to produce the stable amide bond (Scheme 8). Amidation allows the incorporation of important functional moieties on the surface of the nanocrystals via the amide linkage that can be exploited for various applications. The most common conditions involve the method of Bulpitt and Aeschlimann [68], with the use of *N*-(3-dimethylaminopropyl)-N′-ethylcarbodiimide hydrochloride (EDC) and N-hydroxysuccimide (NHS), whereby EDC activates the carboxylic acid to form an O-acylisourea or carboxylic ester. Since the O-acylisourea is prone to hydrolysis, the addition of NHS creates a more stable NHS-ester that is resistant to hydrolysis. As the amine is added, the amidation reaction proceeds with less undesired products since NHS is a better-leaving group [69].

Table 2 shows examples of amidation reactions performed on cCNCs, with TEMPO oxidation being the most common way to generate carboxyl groups on the surface of the nanocrystals. An amidation reaction was performed on TEMPO-oxidized cCNCs from cotton linters and sugar beet pulp cellulose using 4-amino TEMPO in the presence of EDC and NHS at neutral pH [69]. The nitroxide moiety on the product was instrumental in characterizing the product through various spectroscopic techniques. It was found that about 30% of the carboxylic groups engaged in the newly formed amide bonds, with a slightly better coupling (31% compared to 26%) for the cotton linter due to a larger crystal size. The 4-amino-TEMPO-modified cCNCs formed stable dispersions in organic solvents such as chloroform, toluene, and dimethylformamide. Amidation at the reactive carboxyl acid sites was explored to generate precursors for click chemistry [70]. TEMPO-oxidized cCNCs were first modified with 11-azido-3,6,9-trioxaundecan-1-amine through a carbodiimide-mediated reaction to produce terminal azide functionalized CNCs. In a separate reaction, the amino-functional group on propargylamine was coupled with the cCNCs to yield CNCs with a terminal alkyne functionality. The resulting terminal azide and alkyne CNCs derivatives were then coupled via a Cu(I)-catalyzed Huisgen 1,3-dipolar cycloaddition to produce nanoplatelet gels, which were characterized by various spectroscopy and microscopy techniques. Using a similar strategy, the authors designed photoresponsive CNCs for the click reactions by simply using azido-bearing coumarin and anthracene as their azide precursors [71] to couple the propargylamine-CNC adduct formed [70]. Hemraz et al. reported a mild two-step protecting group-free protocol for the synthesis of aminated CNCs starting from sCNCs [72]. The latter was first oxidized using TEMPO oxidation, after which the TEMPO-oxidized cCNCs were reacted with aliphatic diamines under aqueous conditions to produce CNCs functionalized with terminal amino groups. This approach of functionalizing with bifunctional amines of small alkyl length can serve as a handle for bioconjugation and has been recently used for polymer attachment [73]. The cCNCs have also been coupled to aromatic amines [74], resulting in aromatic amides. Due to the hydrophobic nature of these derivatives, these nanomaterials can be dispersed in organic solvents. Amidation reactions have also been performed on cCNCs generated using the APS process to functionalize the nanocrystals with decylamine, dioctylamine, 2-aminoanthracene, and avidin [20].

### 3.2. Esterification

**Scheme 9:**
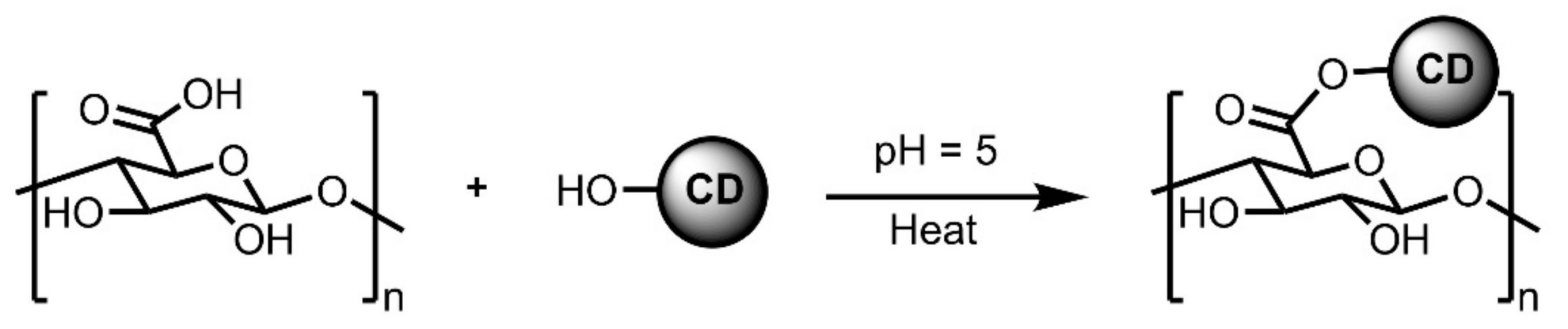
Esterification reaction between cyclodextrins and TEMPO-oxidized cCNCs [82].

Esterification is a common reaction performed on CNCs due to the abundance of primary alcohols on the surface, resulting in the reaction of hydroxyl groups from CNCs with carboxyl acids [10]. The presence of the carboxylic acid group on the surface of cCNCs still allows the scope for esterification via coupling with alcohols. This approach was used to design smart biobased materials for food packaging using cyclodextrins [82]. cCNCs were first prepared through a TEMPO-mediated oxidation reaction. The resulting product was then cast into a sheet and esterified with betacyclodextrin (β-CD) and hydroxypropyl-beta-cyclodextrin (HP β-CD) at 70 °C (Scheme 9). The formation of the ester bonds was confirmed by FT-IR, which showed adsorption bands at around 1730 cm^−1^ and the CD content was determined by phenolphthalein colorimetry. The small molecules, carvacrol and curcumin, were then entrapped by the β-CD and HP β-CD modified cCNCs and their release from the complexes was investigated. It was found that the loading amount of carvacrol and curcumin was significantly increased by the presence of CDs when compared to the complexation of the small molecules with the cCNCs. A prolonged release of carvacrol and curcumin was also observed. In addition, the carvacrol-loaded HP β-CD modified cCNCs displayed excellent antibacterial activities and could have applications as antibacterial products in packaging.

## 4. Applications of cCNCs

The number of applications utilizing cCNCs as an alternative material over the predominant sCNCs has increased in the past decade as the methods for cCNCs’ production mature (Section 2), and researchers have begun to take advantage of both the inherent physicochemical properties and the potential to further tailor the material by surface modification at the carboxyl group (Section 3). Some of these applications will be discussed in detail. Naturally, as a reinforcing material, cCNCs have been used in polymer composites. However, with the biocompatibility of CNC, some of these applications have been directed towards the food and medical sector. The rich surface chemistry and nanoscale structure also enable cCNC to be a structural support for the fabrication of novel chemical and biocatalysts and materials for environmental remediation.

### 4.1. Nanocomposites

CNCs have been used to form nanocomposites due to numerous properties, namely, nanometer dimensions, low toxicity, biocompatibility, biodegradability, high aspect ratio, high Young’s modulus, high strength, renewability, and abundance. Ever since the use of CNCs in polymer matrices was reported in 1995, there have been numerous reports of CNCs in nanocomposites [6,83]. This has also led to the application of cCNCs as components for nanocomposites, specifically utilizing the carboxyl group of the cCNCs to add functional groups that impart specific properties to the whole composite. For example, cCNCs have been used to create organic-inorganic nanomaterials through the carbodiimide-assisted coupling of the polyhedral oligomeric silsesquioxane (POSS) onto the carboxylic acid functional groups. POSS was found to be evenly distributed on the TEMPO-oxidized cCNCs, consistent with covalent bonding along the oxidized surface of C6 groups. However, for sodium periodate-oxidized cCNCs, POSS was found aggregated at different sites of the cellulose chain at either the more crystalline, central regions of the cellulose (where the carboxylic acid groups are located on the C2 and C3) or the oxidized amorphous end chains. Not surprisingly, the thermal stability of all the organic–inorganic nanomaterials increased compared to the starting cCNCs as POSS can impart flame-retardant properties to composites [84].

Thermo-responsive polymers such as Jeffamines [75] and PNIPAM [76] have been grafted onto the surface of CNCs. These polymers can either be hydrophilic below the LCST or hydrophobic above the LCST just by changing conformation of the polymer chains from coil to globule state. Jeffamines are commercial polymers formed from the statistical copolymerization of ethylene oxide and propylene oxide that possess thermosensitive properties. Grafting of thermosensitive amine-terminated Jeffamine polymer chains on the surface of TEMPO-oxidized cCNCs was achieved using a “grafting-onto” strategy, and the resulting materials were characterized by various spectroscopy and microscopy techniques. The loss in electrostatic stability was reflected by the zeta potential measurements. In water, the zeta potential of the TEMPO-oxidized cCNCs was −23.5 mV, and upon grafting, as expected, the potential values decreased in magnitude in the range of −8 to −5 mV. Yet, despite losing on electrostatic stability, the samples did not flocculate, as they gained steric stability from the polymer chains. Through DLS experiments, which showed an increase in hydrodynamic diameter as the temperature was increased, the UV-vis spectra showed a surge in optical density above the LCST. This change in turbidity was reversible with a 4 °C hysteresis effect [75]. While in the case of sCNCs, surface modification was conducted via different polymerization approaches through the primary hydroxyl sites, attachment of PNIPAM chains in this work occurred through the covalent bonds formed between the amino groups of PNIPAM and the carboxylic acid groups of the cCNCs [76]. The TEMPO-oxidized cCNCs had a DO of 0.25, and the percentage of carboxylate groups that were found to have been substituted by amino groups was 30%. The removal of the unreacted polymer chains was ensured by quenching the reaction at a pH of 1–2 and subjecting the resulting product to dialysis. It was found that the polymer chains in PNIPAM-CNC bestowed steric stabilization in not only aqueous suspensions but also in suspensions of high ionic strengths resulting from the addition of salt, thus preventing flocculation. DLS experiments were used to demonstrate a thermo-reversible aggregation, whereby a low hydrodynamic diameter was observed below the LCST. Above the LCST, aggregation led to a five-fold increase in size. In addition to the thermo-sensitive behavior observed by rheological measurements, an increase in viscosity was observed from 0.008 for the TEMPO-oxidized cCNCs to 40 Pa·s for the grafted polymer. This change in viscosity was significantly higher than what was observed for PNIPAM adsorbed on the surface of the nanocrystals (0.3 Pa·s).

**Scheme 10:**
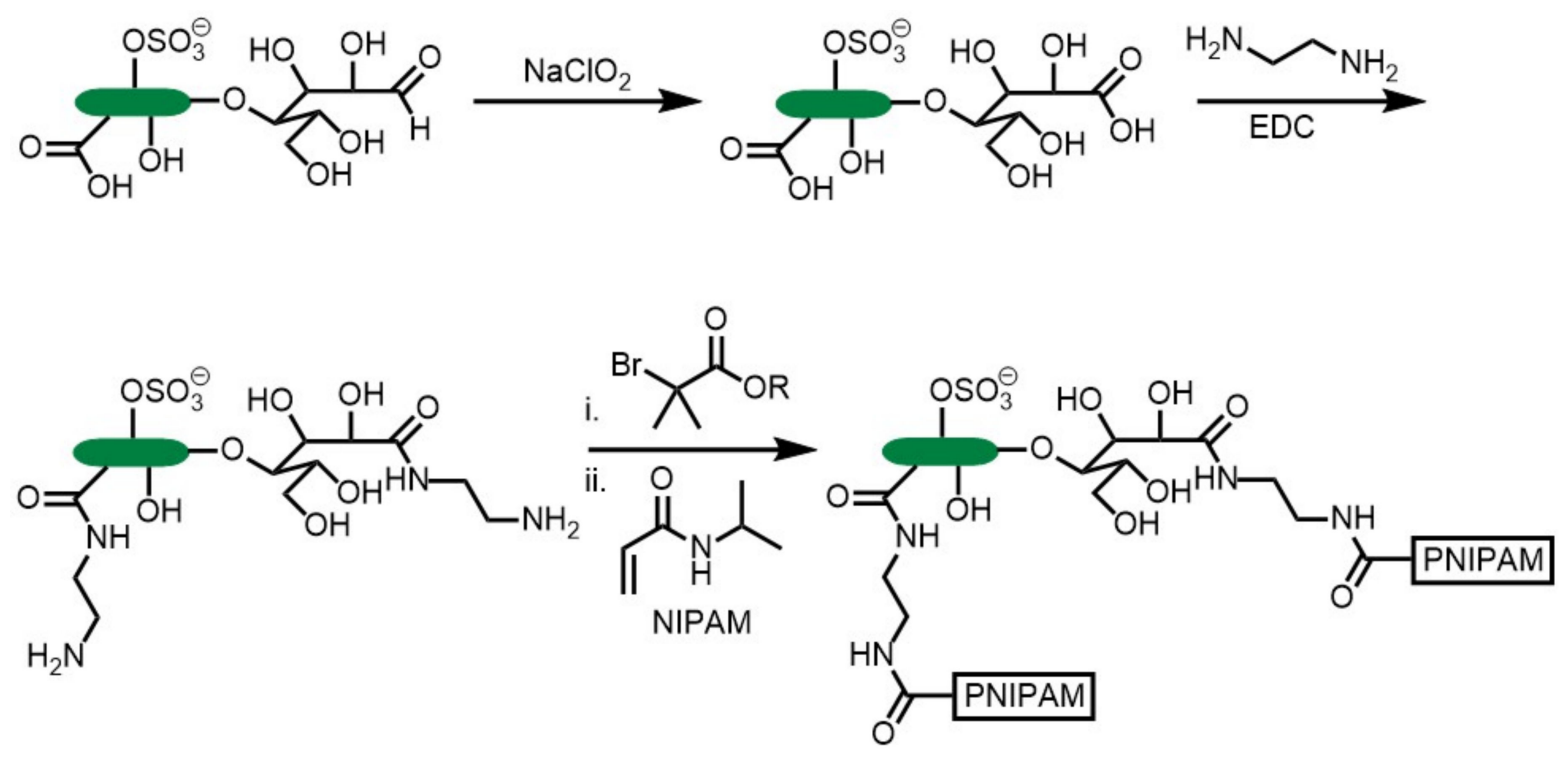
PNIPAM functionalized cCNCs, with preferential attachment at reducing ends of cCNCs [85].

Risteen et al. designed a thermo-responsive poly(N-isopropylacrylamide) (PNIPAM)-based CNC derivative with a thermally switchable liquid-crystalline (LC) phase [85]. PNIPAM has been explored for a wide range of applications, including applications in the biomedical field, since it exhibits a lower critical solution temperature (LCST) at 32 °C. Usually, CNCs with a non-selective attachment of PNIPAM grafts undergo phase separation when heated above the LCST. In this work, the authors conducted a preferential attachment of an atom transfer radical polymerization (ATRP) initiator to the ends of the CNC rods, followed by a surface-initiated ATRP to produce a selectively grafted CNC sample (Scheme 10). The authors used CNCs from USDA, which already contain some carboxyl groups on the surface (26 µmol COOH/g CNCs). The concentration of reducing end groups in the unmodified CNCs was found to be 18.2 µmol CHO per gram of CNCs. The aldehyde functional group at the reducing ends of the CNCs was oxidized using sodium chlorite to produce the carboxylated material at a yield of 57%. The resulting carboxylic acids were then reacted with ethylenediamine to express primary amino groups on the surface of the CNCs. A ninhydrin assay was conducted to determine the concentration of primary amine groups introduced, and it was found to be much higher than expected (60.3 µmol NH_2_/g CNCs). The ethylenediamine molecules did not just covalently react to the carboxylic acids on the surface and ends but also physically adsorbed on the nanocrystals. The aminated CNCs were then subjected to polymerization to produce birefringent materials. Unlike the “brush” PNIPAM-modified CNCs, which have densely packed polymer chains, these selectively grafted CNC have limited polymer chains on the surface, allowing more translational and rotational freedom with minimal attractive interactions. As a result, heating and cooling did not result in phase separation for the selectively grafted CNCs. A similar approach was used to graft thermosensitive polyetheramines at the reducing ends of CNCs [86]. Heating the polymer-functionalized CNC dispersions above the LCST triggered a heat-induced aggregation, leading to the formation of star-shaped assemblies. As the temperature was lowered below the LCST, the assemblies dissociated. This temperature-dependent aggregation and dissociation was reversible and could be monitored by DLS experiments through a change in hydrodynamic diameter. The authors envisage the use of these thermal-responsive CNCs for sensor applications.

Capron and co-workers investigated the modification of bacterial cellulose nanocrystals (BCNs) for the formation of silver nanoparticles [87]. Silver nanoparticles are popular amendments to CNCs as they can confer antimicrobial properties [88]. BCNs were obtained from nata de coco, a jelly-like food produced from the fermentation of coconut water in which *Gluconacetobacter xylinu* secretes microbial cellulose. BCNs exhibit primarily hydroxyl groups, and the researchers modified these nanocrystals to be either cationic (covalent bonding with cholaminchloride hydrochloride), anionic (by TEMPO oxidation), or hydrophobic (EDC-NHS coupling of TEMPO-oxidized cCNCs to octylamine). Native BCNs and the modified BCNs had lengths between 850 and 1750 nm and a width between 20 and 40 nm. The surface charge of native BCNs (−11.2 mV) decreased to −25.4 mV upon TEMPO-oxidation, increased to +15.9 mV in cationic modification, and remained similar in charge when hydrophobically modified (−9.3 mV). Contrary to the assumption that increased negatively charged surfaces would preferentially favor the interaction of Ag^+^ for nanoparticle formation over positively charged surfaces, the key finding was that hydroxyl surface groups on cellulosic surfaces were the real nanoparticle nucleation points for well-defined Ag nanoparticles in all cases. Additional negative surface charges merely improved the dispersion state, thereby increasing the accessibility to the nucleation sites. The ability to form well-dispersed Ag nanoparticles on even hydrophobic BCNs demonstrates the potential to form bifunctional nanocomposite materials in non-aqueous solvents.

The use of self-healing conductive composites with metallic conductivity has been proposed for flexible electronics for enhanced durability and operational lifetime, as these materials are able to restore their structure and functionality from damage [89]. As a biocompatible and hydrophilic polymer, polyvinylalcohol (PVA) composites have been used for electronic skin sensors but suffer from poor mechanical properties, which limits the stretchability of the material [90]. Chen et al. turned to cCNC as a nanofiller to enhance the mechanical properties of flexible films for personal electronics [91]. cCNCs obtained from the hydrolysis of MCC with citric acid were initially coated with polypyrrole (PPy) to form a cCNC-Ppy conductive nano-network, followed by further polymerization with methyl methacrylate (MMA) onto the functionalized CNCs. Ppy is a commonly used, low-cost, conductive polymer, promising conductive polymers with fast impulse discharge performance and vitro/vivo cytocompatibility for personal electronics [92]. PVA was incorporated at different concentrations (1–4%) to the cCNC-Ppy-PMMA composite to form the final conductive, self-healing films characterized by a dynamic network crosslinked by hydrogen bonding. As expected, the addition of cCNCs increased the mechanical strength of the films. However, variations in the concentration of PVA (and their effect on the overall hydrogen-bonding network) also influenced the tensile strength, fracture stress, and fracture strain of the films. As the films were stretched, bent, or deformed, an electric resistance response was obtained, which correlated to the distance of the PPy chains. The self-healing properties of these films were demonstrated by cutting the film and then reheating the cut ends together at 60 °C for 20 min to show that a continuous circuit could be completed to light an LED bulb.

Food packaging has an integral role in the preservation of perishable foods and allows for the optimal use of space during food transport and storage. Most food packaging materials are synthetic polymers that are cheap to produce and offer the required durability and waterproofness required for food preservation. Unfortunately, these polymers are extremely resistant to natural degradation, which leads to significant environmental pollution. There has been an interest identifying biopolymers that can offer similar physicochemical properties to plastics yet are biodegradable and safe for food use. Cao et al. initially developed cassia gum films for edible packaging films assisted by the use of glycerol and sorbitol as plasticizing agents [93]. Cassia gum is a water-soluble polysaccharide extracted from the seeds of the sicklepod consisting of 1,4-ß-D-mannopyranose units and 1,6-linked α-D-galactopyranose units. In these films, they noted their poor mechanical, heat-sealability, and barrier properties. Nanoscale materials have been extensively investigated as potential filler materials in composites for strength reinforcement and impart functionalities, and cCNCs (with dimensions of 100–500 nm in length and 3–10 nm in width) were used in conjunction with glycerol to produce reinforced cassia gum films [94]. The interaction between the cassia gum and cCNCs was based on hydrogen bonding, with no evidence of covalent interactions between the two respective polysaccharides. The inclusion of up to 4% cCNCs to these films resulted in improving the oil permeability, mechanical properties, and heat seal strength compared to the native cassia gum film. At 6% cCNC content, aggregation of the cassia gum and cCNCs was observed.

### 4.2. Biomedical Applications

CNCs are promising materials for biomedical applications due to their hydrophilicity, biocompatibility, their high specific surface area, high aspect ratio, and sharp angles. They have been explored in drug delivery and tissue engineering applications as versatile platforms for protein and enzyme immobilization and in combination with fluorescent probes for bioimaging and biosensing [14]. A fluorescent CNC probe was designed as a potential candidate for nanosensor applications using a 1,8-naphthalimide dye, covalently attached to TEMPO-oxidized cCNCs via an EDC/NHS mediated coupling between the carboxylic acid and amino groups [77]. The resulting reaction was confirmed by FT-IR, UV-vis adsorption, and fluorescence spectra. In addition, no morphological or structural change was observed with the surface-functionalized nanofibers. Since the fluorescence quantum efficiency of dye depends on solvent polarity, the effect of solvent polarity and ionic strength on fluorescent intensity was investigated. It was found that the stronger fluorescence was observed under UV illumination for the dye-labeled CNCs compared to the pure dye. In addition, the colour was enhanced at lower solvent permittivity and higher ionic strength. This behaviour was attributed to aggregation-enhanced emission, resulting from the compression of the electrostatic double layer of the nanocrystals.

Chu et al. went a step further and developed fluorescent nanoprobes by conjugating the amino groups of another 1,8-naphthalimide dye (NANI) and biocompatible poly (ethylene glycol) (PEG) to cCNCs, obtained from the TEMPO/HCl hydrolysis of bleached softwood pulp, through two-step successive grafting [95]. Notably, the addition of PEG brushes on the cCNCs increased the hydrophilicity of the nanomaterial. The resulting materials were investigated in the bioimaging of Hela cells in a physiological environment at high salt concentration. The nanometer dimensions and the morphology were vital in allowing the CNC derivatives to enter and disperse in the cells efficiently. As a result, strong fluorescence emission was obtained in the bioimaging. Nanoprobes consisting of only cCNCs and NANI were found to aggregate within the cell, leading to lower fluorescence emission, but nanoprobes with cCNCs, NANI, and PEG were found to be better dispersed within the cell, leading to greater fluorescence emission.

Rojas and co-workers coupled amino-functionalized carbon quantum dots (CQD) with TEMPO-oxidized cCNCs to produce biocompatible and photoluminescent hybrid material, which was used as a bioimaging probe to image HeLa and RAW 264.7 macrophage cells [78]. The resulting material was characterized using X-ray photoelectron spectroscopy, fluorescence spectroscopy, electron, and confocal microscopies. Unlike the cCNC, the CQD-cCNC hybrid material displayed a green fluorescence upon excitation using UV light (365 nm). It was found that the quantum dots improved the cytocompatibility and internalization of the cCNCs in the cells.

**Scheme 11:**
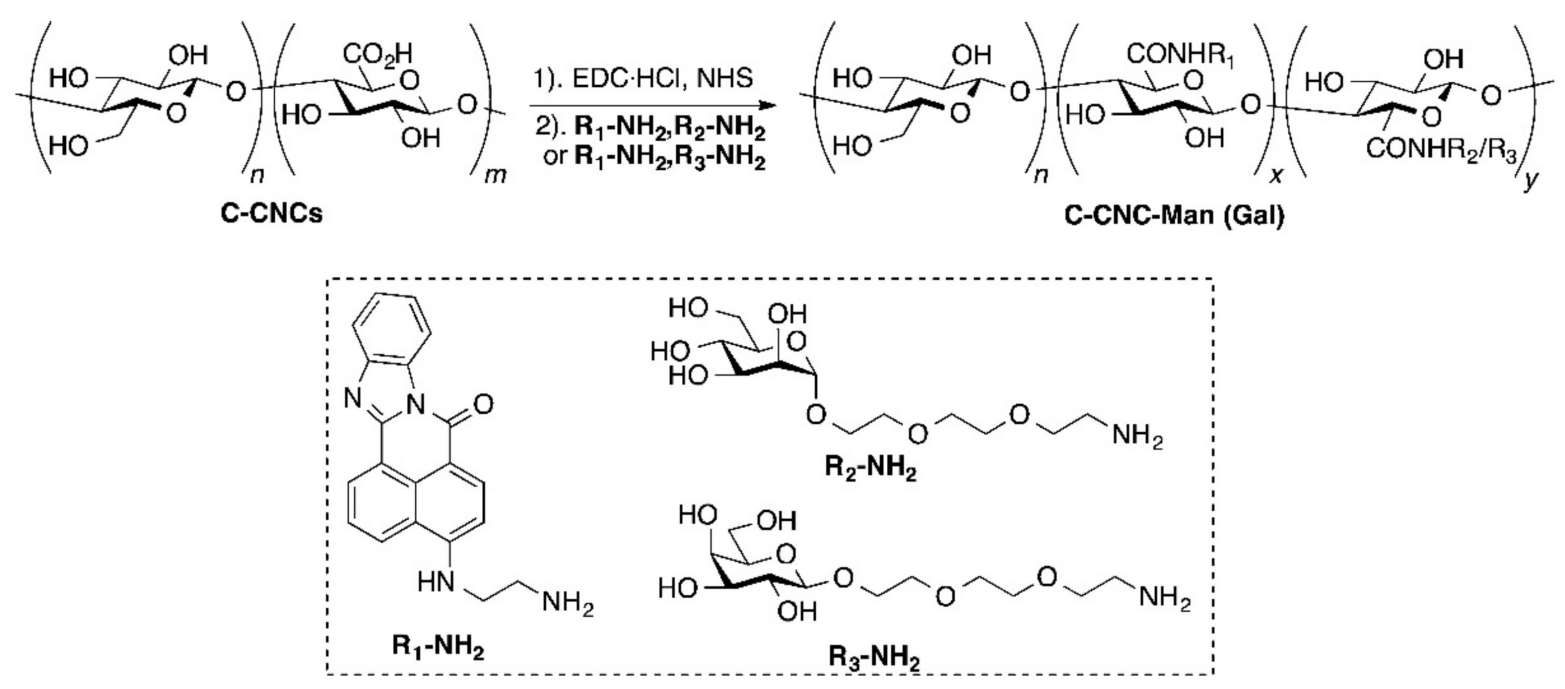
Synthesis of carbohydrate-functionalized and fluorescent cCNCs (Reproduced with permission from [79], Copyright: American Chemical Society 2015).

A multifunctional CNC platform constituting carbohydrate and fluorophore components was developed for lectin recognition and bacterial imaging [79]. The carboxylic acid groups of the TEMPO-oxidized cCNCs were coupled with the amino groups on the fluorescent dye 4-(2-aminoethylamino)-7H-benz[de]benzimidazo[2,1-a]isoquinoline-7-one and the carbohydrate ligand 1-(2-(2-(2-aminoethoxy)ethoxy)ethoxy-D-mannopyranoside via the amide linkage, in the presence of EDC and NHS (Scheme 11). As evidenced by scanning transmission electron microscopy (STEM) images, these dual-functionalized CNCs maintained a rod-like morphology, with an average length of 265 ± 80 nm and width of 5.2 ± 0.3 nm. They also displayed a CrI of 65% and a yellow-green fluorescence upon excitation at 450 nm. DLS was used to investigate the change in the apparent size due to aggregation from lectin binding. A significant increase in hydrodynamic diameter was observed from about 200 nm for the cCNCs to around 400–600 nm, which could be attributed to cross-linking between the lectins and the carbohydrate-functionalized CNCs. These results were supported by transmission electron microscopy (TEM) measurements, which showed agglomeration of the modified CNCs and the protein concanavalin A. Confocal fluorescence microscopy was used to evaluate the biorecognition and binding of the dual-functionalized CNCs to concanavalin A through interactions with the mannose moiety and it was found that mannosylated nanocrystals underwent selective interactions with FimH-presenting *E. coli*.

**Scheme 12:**
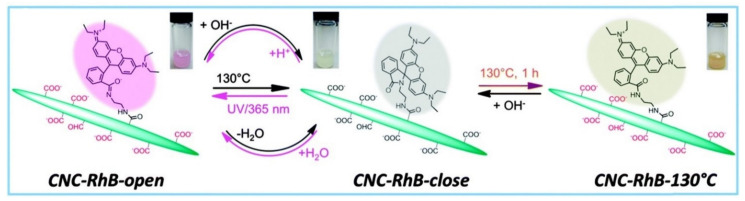
Synthesis of rhodamine-functionalized fluorescent cCNCs (Reproduced with permission from [80], Copyright: Royal Society of Chemistry 2014).

Zhao et al. immobilized rhodamine spiroamide groups on the surface of CNCs in an attempt to design smart materials [80]. The rhodamine B dye was first coupled with ethylenediamine to introduce amino groups to the dye. The resulting aminoethyl rhodamine was then reacted with TEMPO-oxidized cCNCs by coupling the carboxylate groups of the nanoparticles to the primary amino groups on the dye. Elemental analysis was used to determine the amount of aminoethyl rhodamine immobilized on the surface of the nanocrystals, and it was estimated to be about 0.2 mmol/g. These functionalized materials (CNC-RhB) were responsive towards multiple stimuli and underwent a color-switching process as a result of a ring-opening and -closing process initiated by the various triggers applied. As seen in Scheme 12, CNC-RhB displayed a beige color in DMF at neutral pH and room temperature, and subsequent heat treatment at 130 °C led to a ring-opening reaction upon which the color transitioned to yellow. The ring-opening mechanism of the rhodamine B is reversible with the addition of NaOH, which restores the temporary binding between carboxyl groups and rhodamine. Similarly, UV-illumination under acidic conditions produced a magenta-colored solution. These materials have potential applications as smart switchable devices due to their excellent switching performance and reversibility under multiple stimuli. It was also possible to study the structural changes by nuclear magnetic resonance through dynamic nuclear polarization.

Biomaterials have attracted considerable interest in tissue-engineering applications due to their hydrophilic properties and biocompatibility to mimic components of the extracellular matrix. Challenges in the development of artificial extracellular matrix materials include adequate engagement of cell attachment, proliferation, migration, and differentiation, and their ability to transport biomolecules and waste metabolites from native tissue. Recently, extrusion 3D printing of cell-free or cell-loaded hydrogel ink has led to the production of desired compositions and architectures for tissue-engineering applications [96]. However, most biomaterials on their own lack the inherent mechanical properties required to maintain their structural integrity and support stress factors in an in vivo 3D environment [97]. Kumar et al. developed stable cell-free and cell-loaded hydrogel inks for direct-write extrusion-based 3D printing using cCNCs and xanthan gum within a sodium alginate hydrogel matrix [98]. On their own, each biopolymer component lacks the necessary physicochemical properties to produce sufficient bioinks for 3D printing. Sodium alginate has fast gelling properties but inadequate mechanical properties and biocompatibility to interact with the extracellular matrix. Xanthan gum features highly pseudoplastic behavior, good thermal properties, and variable viscosity influenced by shear rate, but poor mechanical properties. The cCNCs (prepared from MCC using APS) were selected specifically for both their reinforcement properties as a nanofiller, and to access their carboxylic acid functional groups for extra crosslinking to the hydrogel network for improving the mechanical properties and shear-thinning behavior during extrusion. Utilizing all three biopolymers, the bioink featured good rheological properties, post-printing fidelity, and dynamic mechanical properties of the gel with viability towards human skin fibroblast (CCD-986Sk) cells.

Lastly, CNCs have been applied towards the formation of Pickering emulsions [99], as they can self-assemble at the oil-water interface due to negative surface charges, wettability, crystalline structure, and morphology. Pickering emulsions can be utilized in a number of biomedical applications, including drug delivery and scaffolding material [100]. Their properties have been Capron and co-workers investigated the ability of sCNCs to form Pickering emulsions and found that samples with the higher negative surface charges produced more stable emulsions [101]. Unlike the fairly labile sulfate half-esters produced during the hydrolysis process, the presence of carboxylate groups from oxidation of the CNCs increased the magnitude and stability of surface charges on the crystals. Surface modification of the oxidized nanocrystals through the ionic exchange of the sodium ions with stearyltrimethylammonium chloride produced CNCs decorated with quaternary ammonium salts, which could form more stable emulsions and larger droplets. Mikulcová et al. investigated APS-oxidized CNCs in the formation of Pickering emulsions from acidic to neutral pH with a triglyceride oil (e.g., tricaprine or tricapryline) [102]. The size of emulsion droplets was dependent on oil and cCNC loading. The authors noted that lowering the pH did not trigger the release of oil from the micron-sized Pickering emulsions, which they attributed to the strong absorption of cCNCs towards the polar triglyceride oil used.

### 4.3. Biocatalysis

In biocatalysis, enzymes are often immobilized on scaffold materials to improve their stability and increase their activity over a free and unsupported enzyme. Nanoscale carriers are favored because of their high surface area, which can increase enzyme loading and reduce diffusion restrictions, leading to enhanced enzyme activity while promoting greater stability over a free enzyme.

Abouhmad et al. investigated the immobilization of lysozymes on the surface of CNCs and its subsequent impact on antibacterial activity [81]. Lysozyme is an antibacterial enzyme that is effective against Gram positive bacteria, while T4 bacteriophage shows activity against both Gram positive and Gram negative bacteria. Lysozyme, from hen egg white and T4 bacteriophage, were covalently linked to cCNCs and glutaraldehyde-activated aminated CNCs. The latter was prepared by first forming the ammonium salt of cCNCs through neutralization of cCNCs with ammonium hydroxide to improve dispersibility. The neutralized cCNCs were then oxidized to their dialdehyde derivative with sodium metaperiodate, followed by reaction with ethylenediamine and subsequent activation by glutaraldehyde. Incubation of lysozymes with the glutaraldehyde-activated aminated CNCs led to conjugates showed increased antibacterial activity and exhibited enhanced storage stability. The effect of immobilization on antibacterial activity and enzymatic activity was also investigated, and it was found that lysozymes adsorbed on CNCs showed weak activities compared to the lysozyme-CNC bioconjugates formed through covalent immobilization. The activities exhibited by the materials are likely related to their surface properties, as evidenced by the zeta potential measurements. The cCNCs, ammonium salt of cCNCs, and glutaraldehyde-activated aminated CNCs showed zeta potential values of about −54 mV, −42 mV, and +31 mV, respectively, while the zeta potentials of the lysozymes were mildly positive (about +7 to +10 mV). Noncovalent adsorption of the lysozymes onto the CNC surface resulted in negatively charged bioconjugates (about −30 mV), while the covalently bound glutaraldehyde-CNC-lysozyme conjugates formed stable and positively charged colloidal suspensions, with zeta potential values ranging between +37 mV and +43 mV.

Koshani and van den Ven also developed a nanocarrier system using cCNCs for the immobilization of lysozymes [66]. cCNCs were targeted as a potential nanocarrier for enzymes due to their non-toxicity, ease of fabrication and modification, biodegradability, and biocompatibility. The nanomaterials were produced from softwood pulp under two methods: TEMPO-oxidation and Cu-catalyzed H_2_O_2_ oxidation [103]. The carboxyl content of H_2_O_2_-oxidized cCNCs (0.9 mmol/g) was higher than TEMPO-oxidized cCNCs (0.7 mmol/g). The carboxyl groups of H_2_O_2_-oxidized cCNCs enabled the immobilization of the lysozymes by adsorption to achieve an enzyme loading of 240 mg/g by adsorption for an immobilization yield of 73% and an enzyme activity of 62%. In comparison, TEMPO-oxidized cCNCs exhibited an enzyme loading of 550 mg/g by adsorption for an immobilization yield of 98% and an enzyme activity of 73%. When a bioconjugation method employing DMTMM was used to immobilize the lysozyme, an inverse relation was obtained in which the H_2_O_2_-oxidized cCNCs obtained an immobilization yield of 65% and an enzyme activity of 60%, compared to an immobilization yield of 54% and an enzyme activity of 52%. As expected, the lower carboxyl content of TEMPO-oxidized CNCs should result in lower enzyme immobilization due to the necessity for carboxylic groups for bio-conjugation. On the other hand, for adsorption, the authors attribute the differences in enzyme immobilization and activity to changes in the structural conformation of lysozymes, influenced by the varying surface roughness and curvature of the different cCNCs.

### 4.4. Catalysis

The high surface area and available surface functional groups have made CNCs an ideal support material for recyclable catalysts. The most developed approaches for CNC-catalysts are as support materials for metal nanoparticles as they can promote the reduction of metals, but other possibilities include the grafting of organometallic species or surface functionalization to create organocatalysts [104]. The surface chemistry of CNCs can promote greater interaction of substrates with the supported catalytic site for reactions to occur more favorably. The majority of catalyst examples utilize sCNCs; however, a few examples for cCNCs have been reported in the literature. An early example of using APS-oxidized cCNCs in a catalyst application was reported by Luong and co-workers for the reduction of 4-nitrophenol to 4-aminophenol [105]. Gold nanoparticles are often used as catalysts for organic transformation reactions, but an agglomeration of these catalysts in a solution can significantly reduce their catalytic activity. Although gold nanoparticles can be stabilized by immobilization on other support materials, these immobilized catalyses often exhibit weaker catalytic activity compared to the free metal catalyst due to lower accessibility of substrates towards immobilized gold nanoparticles. A nanocomposite was prepared by deposition of pre-formed negatively charged gold nanoparticles (AuNP) onto the surface of a positively charged PDDA-coated cCNCs in which its catalytic activity of AuNP/PDDA/cCNCs was demonstrated in the reduction 4-nitrophenol to 4-aminophenol. Tam and co-workers developed a polyamidoamine (PAMAM) dendrimer-grafted cCNCs (G6 PAMAM) as a support material for gold nanoparticles [106]. PAMAM dendrimers were grafted onto TEMPO-oxidized cCNCs followed by in situ reductions of HAuCl_4_ to form gold nanoparticles with diameters of <20 nm without the need for further addition of a reducing agent. Gold nanoparticle formation was influenced by pH and the concentration of the PAMAM-grafted cCNCs: larger nanoparticles tend to form at pH > 3.3 and at higher concentrations of the functionalized cCNCs. The developed catalyst materials were applied towards the reduction of 4-nitrophenol to 4-aminophenol in which its enhanced catalytic behavior was attributed to smaller gold nanoparticles, and the improved dispersity and accessibility of gold nanoparticles within the PAMAM dendrimer domain. Other reports of nanocomposites made of PANAM dendrimers grafted onto TEMPO-oxidized cCNCs have been applied for the removal of Cu (II) from water solutions with the highest Cu (II) adsorption capacity of 92.07 mg/g at 25 °C [73], and for CO_2_ capture at capacities of 13.31 ± 0.38 mg/g at 25 °C, 9.64 ± 0.60 mg/g at 35 °C, and 9.18 ± 1.27 mg/g at 45 °C, respectively [107].

The carboxyl groups of cCNCs offer sites of reactivity for the covalent immobilization of catalytic transition metal complexes that would normally be soluble and difficult to recover in the reaction media. Heterogeneous dirhodium (II) catalysts based on cCNCs were produced for cycloproponation reactions [108]. Ligand exchange between Rh_2_(OOCCF_3_)_4_ and the carboxyl groups was conducted to incorporate the dirhodium (II) catalyst to the surface of the TEMPO-oxidized cCNCs. Using thermogravimetric analysis, the dirhodium (II) content on the cCNC surface was found to be 0.23 mmol/g of cCNCs. The catalytic performance of Rh grafted-cCNC catalyst was investigated in the cyclopropanation of styrene with ethyl diazoacetate, achieving a yield of 80% in 180 min at room temperature compared to the homogeneous catalyst (Rh_2_(OOCCF_3_)_4_) of 84% in 1 min. Not surprisingly, the authors believe the activity of heterogeneous catalysts was limited by mass transfer and loss of mobility as the Rh catalytic centers are confined on the surface of the cCNC support. However, the benefits of the heterogeneous catalyst reside in its stability against leaching and its reusability associated with the covalent bonding of the Rh to the cCNC and catalyst accessibility from separate binding sites on the surface of the nanocrystal.

Hu et al. immobilized reactive Co (III) salen species onto sCNCs to produce heterogeneous catalysts for the synthesis of cyclic carbonates from propylene oxide and CO_2_ [109]. These catalysts achieved a near quantitative yield in 24 h at low CO_2_ pressure of 0.1 MPa with demonstrated reusability in four reaction cycles. Control experiments showed a 22% yield in the absence of any catalyst, but when sCNCs and TEMPO-oxidized cCNCs were used (without the presence of Co (III) in both cases), the yields increased to 35% and 55%, respectively. The authors believe that the surface-functional groups (sulfate or carboxylic groups) could promote catalytic activity to the system through hydrogen bonding between the epoxide starting material and the CNC.

Ellebrecht and Jones investigated the use of cCNC as a support material for bifunctional heterogeneous organocatalysts [110]. Alkyl diamines were conjugated onto TEMPO-oxidized cCNCs (derived initially from sCNCs) by EDC amide coupling to impart both acid and base characteristics to cCNC surfaces. The catalyst materials were used as acid–base catalysts for the aldol condensation reaction of 4-nitrobenzaldehyde with acetone, with comparable activity to aminosilica organocatalysts. Greater rates of reactions were achieved when the solvent system was switched from a water–acetone mixture to an all-organic system (acetonitrile–acetone) system. The authors also noted that the length of diamines used was influential in decreasing catalytic activity: longer diamines promoted crosslinking between the cCNCs while shorter diamines reduced acid–base cooperativity in the overall organocatalyst system. The authors optimized their work on these acid−base heterogeneous catalysts by specifically tailoring surface species on the cCNCs [111]. The sulfate half-ester content was found to not affect catalysis but was essential to organocatalyst functionalization and dispersion. Crosslinking reduces catalyst activity, and diaminopropane was found to be the most optimal diamine base as this chain length led to the least number of crosslinking events between functionalized cCNCs. By controlling the carboxylic acid to amine ratio, the authors were also able to prepare cCNC catalysts effective for the upgrading of furfural (a key specialty chemical derived from biomass resources) by aldol condensation with acetone with high selectivity towards the furfuryl acetone, outperforming mesoporous silica SBA-15-supported catalysts in both activity and selectivity.

### 4.5. Environmental Remediation

The release of harmful organic pollutants in waste streams poses a major environmental and human health concern. Amongst various pollutants, organic dyes are very worrisome due to their complex aromatic structures, which make them chemically stable and resistant to degradation by heat, light, and oxidants in nature. One of the most cost-effective methods for dye remediation is adsorption. Pinto et al. examined the potential of two positively charged CNCs, namely sCNCs and cCNCs, for the removal of the organic cationic dye, Auramine O (AO) [112]. In head-to-head batch adsorption experiments, the highest removal percentage (82%) and adsorption capacity (20 mg/g) were obtained for the sCNCs for an equilibrium contact time of 30 min. Adsorption behavior of AO on both CNCs was well-described by Freundlich isotherm. The calculated thermodynamics parameters indicated that AO adsorption on both CNC samples was a spontaneous exothermic process. In contrast, while entropy decreased for the sCNCs, an increase in entropy was reported for cCNCs. Overall, the adsorption process of AO dye on the CNC was thought to be driven by two mechanisms: electrostatic interactions between the positively charged nitrogen of AO with anionic sulfate or carboxylate groups of the respective CNC, and hydrogen bonding between the AO tertiary nitrogen atoms with the CNC hydroxyl groups.

Samadder et al. developed magnetic composites based on polyacrylic acid (PAA) crosslinked with acrylic-functionalized Fe_3_O_4_ nanoparticles (M3D) and cCNCs for the removal of the widely used and toxic cationic dye, methylene blue, from aqueous solutions [113]. The cCNCs were extracted from sawdust waste. The latter first underwent stepwise treatments to remove lignin, pectin, and hemicellulose, and the resulting purified cellulose was subjected to sulfuric acid hydrolysis, followed by TEMPO oxidation. The nanopolysaccharide reduced the gel-like properties of the whole nanocomposite particles (60–90 nm in size), which was found to be advantageous for use as an adsorbent. The maximum adsorption capacity increased from 114 mg/g for the M3D–PAA nanocomposite to 332 mg/g for the M3D-PAA-cCNC nanoparticles for methylene blue. These nanoparticles displayed better performance in alkaline conditions as the surface charge of the particles became increasingly negative.

In another remediation report involving methylene blue, Luong and co-workers utilized the high aspect ratio of cCNCs, and the negative surface charges resulting from the carboxylate groups generated via the APS process, to adsorb the toxic cationic dye [114]. It was found that cCNC has a homogeneous surface and a maximum adsorption capacity of 101 mg per gram of nanomaterial, only moderately lower than the maximum adsorption capacity of 127 mg/g for a carbon monolith. Ethanol was 90% effective in desorbing methylene blue from cCNCs using centrifugation. Adsorption and remediation of the dye using cCNCs and ethanol could be a potential method for the removal of organic pollutants. It should be noted that sCNCs from cellulose fibers, as well as its carboxylated derivative obtained from subsequent TEMPO oxidation, also demonstrate adsorption abilities. While the TEMPO oxidation certainly adds an extra step towards the formation of the cCNCs, the adsorption capacity improved from 118 mg to 769 mg of methylene blue per gram for cCNCs [115].

Most of the studies conducted on the adsorption capacity of cCNCs relied on the high aspect ratio of the rod-like nanoparticles. A recent study provided new insights into the ability of cellulose microbeads, made from cCNCs by a hydrogen peroxide method, to adsorb methylene blue [65]. The dye was spray-dried onto the cCNC microbeads, and the adsorption was quantified using a film-pore diffusion model. This work highlights the transport properties of the microbeads and shows how nanoparticles can be rescaled into functional microscale objects for water purification and applications requiring controlled uptake and release.

The removal of salts such as NaCl, Na_2_SO_4_, and MgSO_4_ is an important task with respect to water remediation, particularly for the treatment of brackish water desalination. Salt-rejecting membranes generally require high permeate flux and selectivity, as well as good mechanical properties to purify drinking water [116]. Nanomaterials have been used as additives in membranes to increase their performance and reduce environmental impact. The use of sCNCs as a beneficial addition to polyamide (PA)-polyethersulfone (PES) membranes for enhancing nanofiltration of Na_2_SO_4_ and NaCl aqueous solutions was investigated [117]. The sCNC layer was found to not only control the degree of cross-linking of the formed PA layer but also increased the hydrophilicity of the membranes, allowing for greater water permeation through CNC-containing membranes compared to PES and PA-PES membranes alone. The effects of where the TEMPO-oxidized cCNCs were incorporated in the production of thin-film membranes also consisting of PA and PES for water desalination of Na_2_SO_4_, MgSO_4_, and NaCl was studied [118]. A higher permeate flux was reported when the cCNC was incorporated into the active layer of the membrane. However, when the cCNC was added to the support layer of the membrane, increased salt rejection and mechanical performance were achieved for the thin-film membranes.

**Scheme 13:**
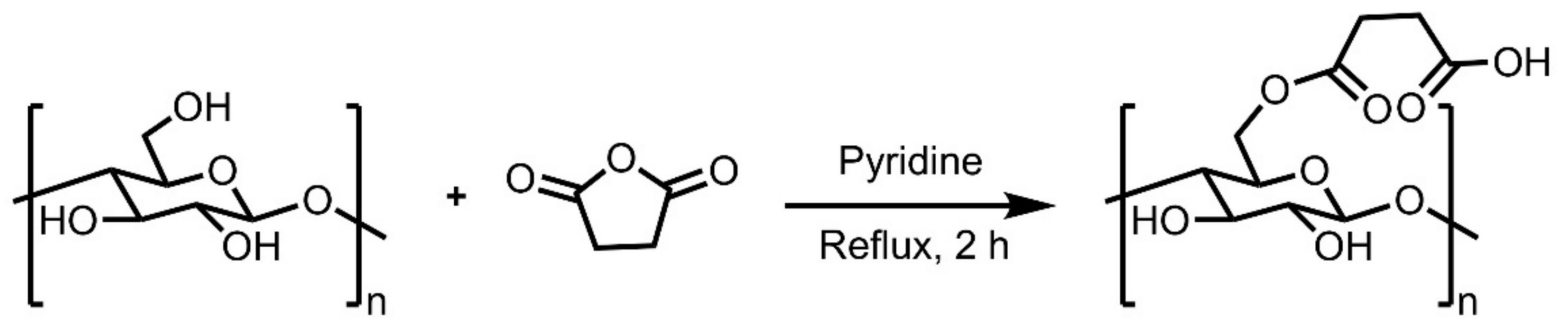
Reaction of sCNCs with succinic anhydride to produce another type of carboxylated CNC [119].

Nanoabsorbents formed from the functionalization of sCNCs with succinic anhydride were prepared for the removal of heavy metal ions (Scheme 13) [119]. The resulting succinic carboxylated CNC (SCNC) was then converted into the sodium form by treatment with a saturated sodium bicarbonate solution and freeze-dried to yield the sodium nanoabsorbent (NaSCNC). Adsorption experiments conducted on SCNC and NaSCNC demonstrated that the nanomaterials were able to adsorb the heavy metal ions Cd^2+^ and Pb^2+^. A higher adsorption capacity was exhibited by NaSCNC due to the ability of the carboxylate to bind more strongly to the nanomaterial than in the acid form. In addition, it was found that adsorption was pH-dependent, with higher adsorption ability for the metal ions at higher pH. The authors believe that at lower pH, more protons are competing with metal ions for the active binding sites. At the same time, the adsorbent surface is positively charged, which increases the difficulty for positively charged metal ions to approach the adsorbent due to electrostatic repulsion. The maximum adsorption capacities of SCNC and NaSCNC were found to be 259.7 mg/g and 344.8 mg/g for Cd^2+^, and 367.6 mg/g and 465.1 mg/g for Pb^2+^, respectively. It was interesting to note that both SCNC and NaSCNC showed high selectivity and binding for Pb^2+,^ and adsorption was not affected by the presence of coexisting ions. NaSCNC was also efficiently regenerated upon treatment with a mild saturated NaCl solution, with no loss of adsorption capacity after two recycles.

Going a step further, Lu et al. designed a magnetic composite made of Fe_3_O_4_ nanoparticles and cCNCs (Fe_3_O_4_-CNC) for adsorption of Pb^2+,^ which did not require filtration or centrifugation to recover the adsorbent [57]. The dicarboxylated CNCs were produced using the oxidative hydrolysis of MCC by APS, followed by esterification with citric acid. With two carboxylic acid moieties per AGU (Scheme 5), the resulting product was co-precipitated with Fe_3_O_4_ nanoparticles to form a composite (Fe_3_O_4_-CNC) with enhanced dispersibility. With the templating effect of the cCNC, the hydroxyl and carboxylic acid groups on the cCNC surface hydrogen-bonded with the hydroxyl groups on the surface of the Fe_3_O_4_ nanoparticles. The Fe_3_O_4_ succeeded in imparting a lower but sufficiently high saturation magnetization for Fe_3_O_4_-CNC (34.13 versus 71.08 emu·g^−1^) to enable fast magnetic separation. Adsorption experiments conducted on Fe_3_O_4_-CNC showed a maximum adsorption capacity of 63.78 mg/g for Pb^2+^ and easy separation from the aqueous solution using magnetism. Dicarboxylated CNCs also improved coagulation and flocculation performance for cationic dyes, kaolin suspension, and textile effluent [120]. The dicarboxylated CNCs, with an approximate dimension of 200–250 nm in length, were generated from a citric acid and HCl hydrolysis of MCC, with a reaction time of 2–6 h and yields of about 80–90%. Compared to the TEMPO-oxidized cCNCs [115], these dicarboxylated CNCs showed a higher adsorption capacity for methylene blue removal. In the presence of the coagulant CaCl_2_, dicarboxylated CNCs displayed remarkable coagulation–flocculation potential by showing a turbidity removal of 99.5% for kaolin suspension. Similarly, dicarboxylated CNCs produced from hydrochloric acid and citric acid treatment of bamboo have also been used as templates to produce zinc oxide-CNC hybrid materials to adsorb within 5 min cationic dyes methylene blue and malachite green at about 91% and 98%, respectively [121]. In addition, the zinc oxide-CNC materials exhibited high antibacterial activities against *E. coli* and *S. aureus*.

### 4.6. Rheology Modifiers

Researchers have turned to cCNCs as novel ingredients for rheology modification with enhanced lubricating and heat-transfer properties. In tribology, a common challenge is to identify lubricants for moving systems that will work well over large temperature ranges and have low-temperature fluidity at nominal viscosity. Nanomaterials such as metal oxides and carbonaceous materials have attracted considerable attention as additives in lubricants due to their inherent properties to decrease wear and friction in moving objects caused by high pressures and temperatures [122]. These nanoparticles can interact better than larger particles to form surface protective films, which contribute to the improvement of anti-wear performances. However, metal oxides themselves are under scrutiny as they can contribute to corrosion and introduce impurities that are considered toxic. Awang et al. investigated cCNC as an additive for SAE40 engine oil and found that 0.1% cCNC exhibited the lowest coefficient of friction (COF) and the strongest wear resistance under all conditions in the piston skirt-liner tribometer test [122]. In the absence of cCNC, worn surfaces showed large areas of metal exfoliation, metal burr, and wear debris. However, with cCNCs in the engine oil, the cCNCs are able to chemically react with surfaces to form a tribo-boundary film that deposits above the frictional surfaces and lower the COF, resulting in minimal surface exfoliation.

The removal of heat is essential for maintaining the longevity of tools that would otherwise wear out and fail due to excessive heat generation. As an alternative to metal oxides, Samylingam et al. investigated the use of cCNC as a sustainable additive for coolant fluid for machining [123]. These fluids consist of cCNCs (ranging in concentration from 0.1 to 1.5%), ethylene glycol, and water. As the concentration of cCNCs increased, the coolant fluid exhibited greater thermal conductivity and viscosity. A coolant fluid of 0.5% cCNC improved tool life through superior heat transfer fluid in comparison to the benchmark metalworking fluid used for lathe machining operation.

**Scheme 14:**
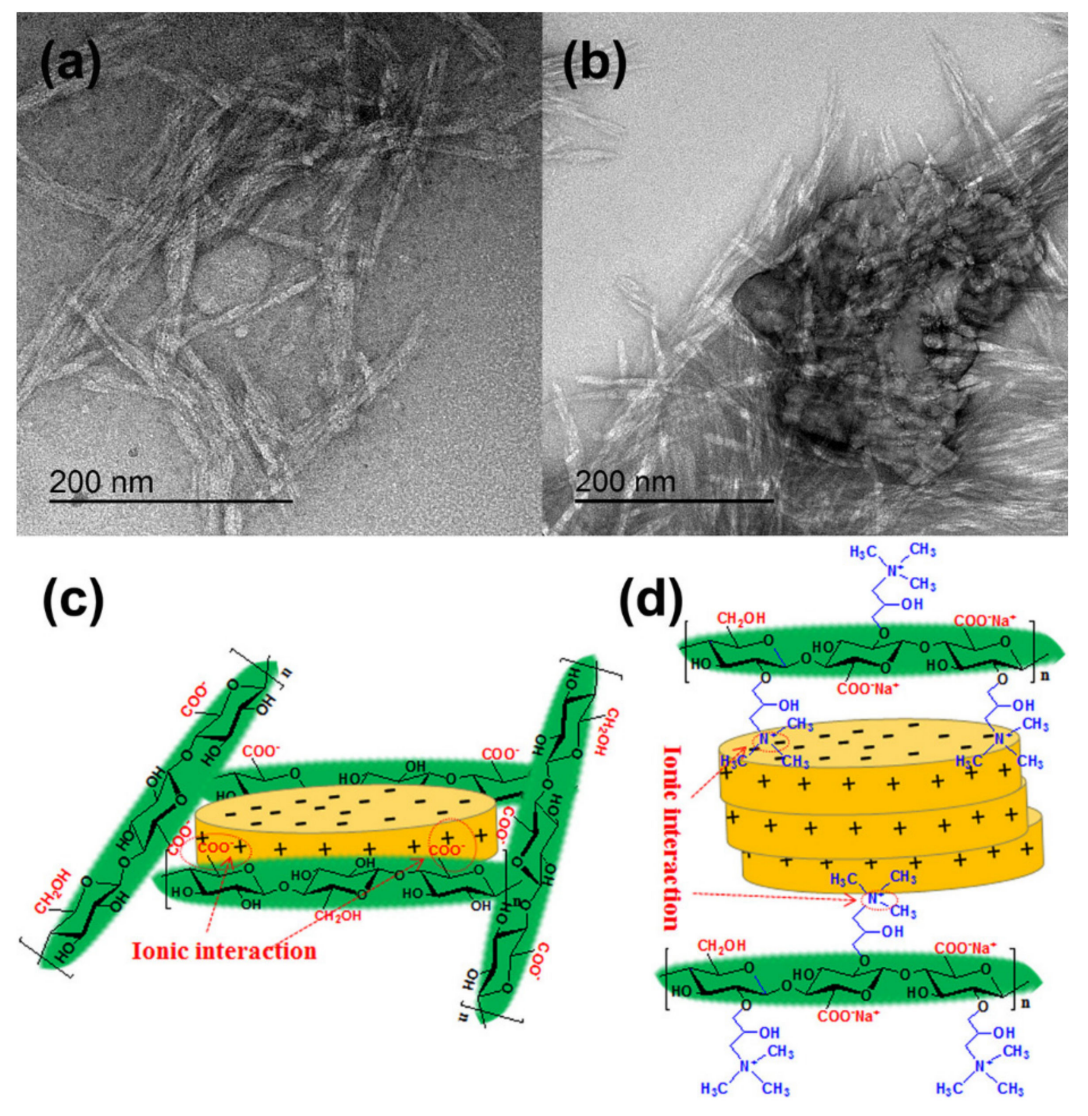
TEM micrographs and proposed surface interactions of water-based drilling fluids formulated using cCNC and bentonite in an edge-to-edge interaction (**a**,**c**) and cationic CNC and bentonite in a face-to-face interaction (**b**,**d**). (Reproduced with permission from [124]. Copyright: American Chemical Society, 2018).

Bentonite is an absorbent swelling clay consisting mostly of montmorillonite. It has attracted interest as suspensions for water-based drilling fluids in oil well excavation but suffers from poor rheology and filtration performance at low solid content. Li et al. compared the use of cCNC and its cationically modified derivative (caCNC-reaction of cCNC with 2,3-epoxypropyl)trimethylammonium chloride) as rheology and filtration modifiers for bentonite suspensions. cCNC (at 0.5% concentration) were found to be better rheological and filtration agents in water-based drilling fluids compared to caCNC [124]. This was directly attributed to how the bentonite suspensions dispersed when cCNCs and cationic cCNCs were added. For native cCNCs, the bentonite platelets were bridged together by the cCNCs in an “edge-to-edge” interaction (Scheme 14). Cationic cCNCs were absorbed on the face surface of bentonite platelets leading to stack layers of platelets in a “face-to-face” interaction. The authors believe the edge-to-edge interaction created by the cCNC favors a more rigid network, thus inducing superior rheological performance over caCNC. In the corresponding patent application, cCNCs were found to be superior to non-carboxylated CNCs as they provide a more uniform dispersion state of the bentonite platelets attributed to the interactions between the carboxyl groups on the nanocrystals and the weakly positively charged edge surfaces of the bentonite platelet [125].

The same group later developed water-based drilling fluids with thermo-controllable rheological properties in response to the need for stimuli-responsive drilling fluids that can maintain rheology based on the ever-changing conditions (i.e., temperature, pH, pressure, etc.) that oil reservoirs exist in. This was accomplished through cCNCs grafting with poly(2-acrylamido-2-methyl-1-propanesulfonic acid) (PAMPS) and PNIPAM by APS free radical polymerization [126]. PAMPS, with its amido functional groups and negatively charged sulfonate groups, promoted the creation of well-dispersed bentonite clusters, while PNIPAM enabled thermo-thickening rheological characteristics for the fluid at elevated temperatures.

## 5. Conclusions and Future Outlook

In this review, we present the known production methods for generating cCNCs. These range from (1) post-production modification of CNCs by TEMPO oxidation, (2) direct conversion of biomass to cCNCs using oxidizers, (3) treatment of cellulosic sources with organic acids, to (4) transformation of cellulose-rich substrates to cCNCs using a mixture of oxidizers, organic acids, and mechanical processing. The presence of the carboxyl groups on the surface of cCNCs provides unique opportunities for the carboxylated material to supersede the predominant sCNCs. Indeed, compared to sCNCs, cCNCs can be easily modified through various chemical transformations at the carboxyl group, while still maintaining their dispersibility. Such surface modification is a gateway to incorporating new functional properties for the design of composites, hydrogels, Pickering emulsions, and films that can be applied to biomedical, agrochemical, and electronic domains. Other less-explored opportunities reside in the use of cCNCs as scaffold materials for enzymes and catalysis.

Large-scale utilization of sCNCs is only beginning to take place, which has necessitated the need for ton-scale production of sCNCs. The current cCNCs production is only at a pilot scale, and it is likely that cCNCs will face similar challenges in identifying anchor applications to justify upscaling the production of the nanomaterial. Feedstock availability, batch-to-batch consistency, and the reduction of water and chemical consumption are critical barriers to the commercial production of cCNCs that need to be overcome. Most current processes utilize an exorbitant amount of acids and oxidizers; therefore, efforts should be focused on making cCNCs under milder and greener reaction conditions. This could entail developing enzymes that could selectively attack the amorphous regions of cellulose and transform the primary hydroxyl groups into carboxyl groups. Enabling the recycling of reagents would also help in reducing the detrimental environmental impact of these oxidative processes. Finally, little is known on the environmental impact of CNCs as there is no information available on the fate of these entities in the environment nor on life-cycle analysis [127]. Efforts aiming at addressing these pertinent environmental questions are necessary for all types of CNCs.

## Figures and Tables

**Table 1:** Production of cCNCs from different feedstock materials.

Feedstock	Method	Length (nm)	Width (nm)	CrI (%)	Yield (%)	Carboxyl Content (mmol/g)	Ref.
Cotton linters	HCl, TEMPO	n.r.	4–5	75	81	0.15 (DO)	[22]
Sugar beet pulp	HCl, TEMPO	n.r.	3–4	52	63	0.23 (DO)	[22]
Softwood-bleached kraft pulp	TEMPO, cavitation	155–244	3.6	81	94	1.57	[24]
MCC	TEMPO, cavitation	163–192	4.3	88	70	1.12	[24]
Flax fibers	APS	144 ± 5	3.8 ± 0.1	75	28	0.18 (DO)	[20]
Flax shives	APS	296 ± 16	5.1 ± 0.1	64	22	0.19 (DO)	[20]
Hemp fibers	APS	148 ± 3	5.8 ± 0.1	73	36	0.17 (DO)	[20]
Triticale straw extract	APS	134 ± 5	4.2 ± 0.1	73	31	0.11 (DO)	[20]
MCC	APS	128 ± 4	5.5 ± 0.1	83	65	0.19 (DO)	[20]
Whatman CF1	APS	121 ± 3	6.7 ± 0.3	91	81	0.12 (DO)	[20]
Wood pulp	APS	124 ± 6	6.0 ± 0.2	81	36	0.19 (DO)	[20]
Bacterial cellulose	APS	88 ± 5	6.5 ± 0.2	70	14	0.14 (DO)	[20]
Black spruce dissolving pulp	H_2_O_2_	170	10	n.r.	n.r.	0.15	[21,65]
Dissolving pulp	NaOCl, transition metal catalyzed	100–150	9–14	80	n.r.	0.15	[49]
Lemon seed	APS	140–160	10–20	74	13	n.r.	[41]
Lemon seed	TEMPO	340–380	26–42	66	52	n.r.	[41]
Juncus plant	Citric acid/HCl	352 ± 79	6.1 ± 2.8	83	n.r.	n.r.	[58]
Tomato plant residues	Citric acid/HCl	514 ± 131	4.7 ± 1.4	78	n.r.	n.r.	[59]
Bamboo borer powder	APS	20–50 (spheres)	-	63	n.r.	n.r.	[42]
Recycled paper	APS	130 ± 15	4.7 ± 2.5	72	22	0.57	[40]
Tunicate	APS	383 ± 164	10.9 ± 2.3	86	n.r.	0.07 (DO)	[44]
Cotton powder	APS, microwave	80–400	7	n.r.	46	n.r.	[39]
Softwood pulp	TEMPO	103 ± 29	21 ± 7	n.r.	12	0.7	[66]
Softwood pulp	Cu_2_SO_4_/H_2_O_2_	247 ± 58	6 ± 2	n.r.	51	0.9	[66]
Cotton pulp	APS/TMEDA	80–350	3–12	91	63	1.38	[37]
Cotton pulp	H_2_SO_4_, KMnO_4_/oxalic acid	150–300	10–22	89	68	1.58	[51]
MCC	H_2_SO_4_, HNO_3_	186 ± 13	9 ± 3	91	24	0.11	[52]
Sugarcane bagasse	Citric acid, ultrasonication	251 ± 52	21 ± 6	77	32	0.60	[55]
MCC	Citric acid, HCl	231.8 ± 23.2	15.8 ± 3.0	83	88	1.40	[56]
Bleached eucalyptus kraft pulp	Citric acid, FeCl_3_	100–250	6–12	80	80	0.90	[60]
Bleached eucalyptus kraft pulp	Oxalic acid	278	19	81	25	0.19	[53]
Softwood pulp	Sodium periodate, sodium chlorite	120–200	13	91	n.r.	6.6	[61]
Bleached kraft pulp	H_2_SO_4_, sodium chlorite, 4% hypochlorite	150–200	5–20	n.r	50	n.r	[67]
Elephant grass	TEMPO, ultrasonication	174 ± 73	5.2 ± 1.4	n.r	n.r	n.r.	[32,33]
Bleached kraft pulp	LiBr/H_2_SO_4_, APS	57 ± 24	9.3 ± 3.1	96	52	0.44	[45]
Recycled medium-density fiberboard	APS	170–365	13–17	63	55	0.10 (DO)	[46]
Denim waste	NaOH, APS	81.5 ± 20.5	18.4 ± 2.5	27	79	n.r.	[47]
Balsa fibers	APS	95.5	6.5	n.r.	57.7	n.r.	[48]
Kapok fibers	APS	112.1	5.8	n.r.	60.7	n.r.	[48]

n.r. = not reported; DO: degree of oxidation.

**Table 2:** Amidation reactions on cCNCs for the design of functionalized CNCs in various applications.

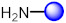	Reaction Conditions	Dispersion	DO of Ccnc ^a^	Application	Reference
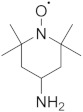	EDC, NHS, pH 7.5–8 24 h, 50 °C	CHCl_3_, THF, toluene	0.21	Nanocomposites	[69]
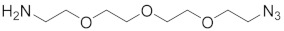	EDC, NHS, MES pH 4, 24 h, RT	Ionic liquid	0.2–0.28	Click chemistry Nanoplatelet gels	[70]
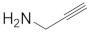	EDC, NHS, MES pH 4, 24 h, RT	Ionic liquid	0.2–0.28	Click chemistry Nanoplatelet gels	[70,71]
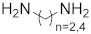	EDC, NHS, MES pH 5, 24 h, RT	Aqueous	0.25	Conjugation	[72]
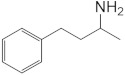	EDC, NHS, pH 8–8.5 72 h, RT	Acetone CH_2_Cl_2_	0.22	Nanocomposites	[74]
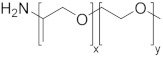	EDC, NHS, pH 7.5–8 24 h, RT	DMF/aqueous	0.2	Thermo-responsive materials	[75]
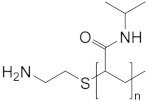	EDC, NHS, pH 7.5–8 48 h, RT	Aqueous	0.25	Thermo-responsive materials	[76]
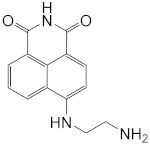	EDC, NHS, pH 7.2, PBS, DMF16 h, RT	DMF/aqueous	1.52 ^b^	Nanosensors	[77]
Quantum Dots-NH_2_	EDC, NHS, pH 5, overnight, RT	Aqueous	0.24	Bioimaging	[78]
Amino-functionalized sugars	EDC, NHS, MES pH 7.5–8.5 16 h, RT	Aqueous	1.56 ^b^	Biorecognition	[79]
Fluorescent quinoline dye	EDC, NHS, MES pH 7.5–8.5 16 h, RT	Aqueous	1.56 ^b^	Bioimaging	[79]
Aminoethyl rhodamine dye	CDI, DMF, 20 h, 60 °C	DMF/aqueous	1.102 ^b^	Molecular switching, imaging	[80]
Lysozyme-NH_2_	EDC, NHS, MES pH 5.7, 3 h, RT	Aqueous	-	Antibacterial	[81]
Lysozyme-NH_2_	DMTMM, 1 h, 37 °C	Aqueous	0.7–0.9 ^b^	Emulsifier	[66]

^a^ The values displayed for the DO or carboxyl content of cCNC correspond to values prior to the amidation reactions; ^b^ Carboxyl content (mmol/g); EDC: *N*-(3-dimethylaminopropyl)-N′-ethylcarbodiimide hydrochloride; NHS: N-hydroxysuccimide; MES: 2-(N-morpholino)-ethanesulfonic acid buffer; DMTMM: 4-(4,6-dimethoxy-1,3,5-tr,azin-2-yl)-4-methylmorpholinium chloride; CDI: 1,1′-carbonyldiimidazole.
